# Founder cell configuration drives competitive outcome within colony biofilms

**DOI:** 10.1038/s41396-022-01198-8

**Published:** 2022-02-05

**Authors:** Lukas Eigentler, Margarita Kalamara, Graeme Ball, Cait E. MacPhee, Nicola R. Stanley-Wall, Fordyce A. Davidson

**Affiliations:** 1 Division of Molecular Microbiology, School of Life Sciences, University of Dundee, Dundee, DD1 5EH UK; 2 Mathematics, School of Science and Engineering, University of Dundee, Dundee, DD1 4HN UK; 3 Dundee Imaging Facility, School of Life Sciences, University of Dundee, Dundee, DD1 5HN UK; 4 School of Physics and Astronomy, The University of Edinburgh, Edinburgh, EH9 3FD UK

**Keywords:** Biofilms, Theoretical ecology

## Abstract

Bacteria can form dense communities called biofilms, where cells are embedded in a self-produced extracellular matrix. Exploiting competitive interactions between strains within the biofilm context can have potential applications in biological, medical, and industrial systems. By combining mathematical modelling with experimental assays, we reveal that spatial structure and competitive dynamics within biofilms are significantly affected by the location and density of the founder cells used to inoculate the biofilm. Using a species-independent theoretical framework describing colony biofilm formation, we show that the observed spatial structure and relative strain biomass in a mature biofilm comprising two isogenic strains can be mapped directly to the geographical distributions of founder cells. Moreover, we define a predictor of competitive outcome that accurately forecasts relative abundance of strains based solely on the founder cells’ potential for radial expansion. Consequently, we reveal that variability of competitive outcome in biofilms inoculated at low founder density is a natural consequence of the random positioning of founding cells in the inoculum. Extension of our study to non-isogenic strains that interact through local antagonisms, shows that even for strains with different competition strengths, a race for space remains the dominant mode of competition in low founder density biofilms. Our results, verified by experimental assays using *Bacillus subtilis*, highlight the importance of spatial dynamics on competitive interactions within biofilms and hence to related applications.

## Introduction

Biofilms are consortia of microorganisms [1]; cells embedded in a self-produced extracellular matrix typically comprising extracellular polysaccharides, proteins, DNA, and components of lysed cells [1–5]. Many natural, industrial, and medical environments are significantly affected by biofilm formation. For example, biofilms are used in wastewater treatment [6], are fundamental to the functioning of microbiota in the human gastrointestinal tract [7], and are central to global biogeochemical cycling [8, 9]. However, from a human perspective, the role of biofilms is not exclusively positive, as they are a known cause of the majority of chronic infections [10] and fouling of medical devices [11].

Many in vitro methods have been developed to study diverse biofilms. The complexity of the assay set-up, and the ability of each system to mimic physiologically relevant conditions is highly varied [3]. One simple, but widely used method is the colony biofilm assay. In this system, founding cells are deposited on an agar-solidified growth medium and the architecturally complex macroscale structure that develops is easily examined [3]. The colony biofilm assay has proven effective in revealing regulatory pathways involved in controlling biofilm formation and the production of molecules found in the biofilm matrix. Hence, it has been implemented widely across many different microorganisms [1, 3, 12].

Increasingly, the colony biofilm assay is being exploited to explore competitive dynamics within single and mixed species biofilms [13–19]. Antagonisms, here defined to comprise any interaction mechanism that causes a reduction in net growth of a target strain, are common within multi-strain biofilms [13]. Many antagonistic mechanisms fundamentally depend on spatial co-location of strains. Therefore, spatial segregation (i.e., the separation of genotypes in space) within multi-strain biofilms offers protection [14, 17, 20, 21]. Hence, a necessary precursor to a full understanding of the effects of local antagonisms on competitive dynamics within biofilms is a comprehensive understanding of the impact of spatial dynamics. Spatial segregation in biofilms can be induced artificially by the careful deposition of founder cells used to inoculate the biofilm in a prescribed spatial structure, using ‘cell printing’ methods [20, 22–25]. However, spatial segregation within multi-strain biofilms can also arise from well-mixed founder cells, particularly if the density of founder cells is low [14, 16, 17, 21, 26]. The observed increase of spatial segregation with decreasing founder density is typically attributed to increased spatial separation of the founder cells. The hypothesis is that this initial separation allows for the establishment of distinct, single-strain microcolonies, before these expand and encounter each other [14, 17]. However, despite a growing body of work in this area, the mechanisms by which competition for space is affected by the location and density of founder cells are still unclear.

Here, we elucidate the fundamental role of spatial dynamics in single- and dual-strain colony biofilms across a wide range of founder densities. We combine mathematical modelling with an experimental co-culture colony biofilm assay using *Bacillus subtilis*, a well-understood Gram-positive bacterium. We first focussed on single-strain biofilms to restrict competitive dynamics between founder cells to those related only to space. We mapped parts of the mature biofilm to founder cells and showed that the random placement of founder cells significantly affected the spatial structure of the mature biofilm. This analysis led to the development of an accurate predictor for competitive outcome. We showed that this predictor remained accurate in a study of dual-strain biofilms, in which the net growth rates of the strains were reduced due to local antagonisms. Implementing the predictor revealed that competition for space remained the dominating mode of competition, even for strain pairs in which the strengths of local antagonistic interactions were disproportionally skewed towards one strain.

## Results

### A theoretical framework of interacting bacterial strains

**Fig. 1:**
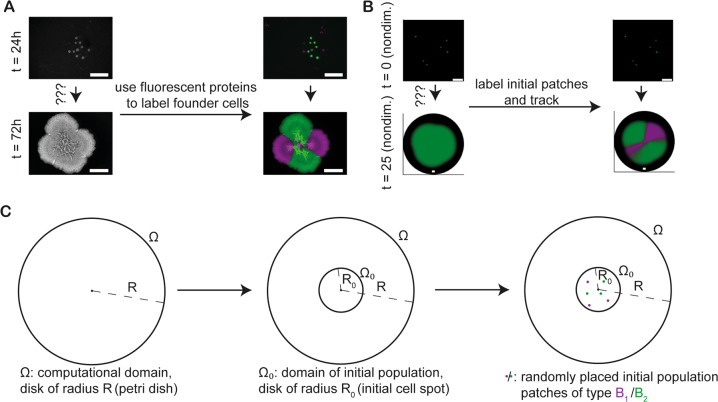
Experimental and modelling set-up. **A** An example of the experimental assay. Founder cells carry either a constitutively produced copy of GFP (green) or mTagBFP (magenta). The bacteria were mixed in a 1:1 ratio and images taken after 24 h and 72 h of incubation. The number of founder cells was approx. 10 CFUs. The scalebars are 5 mm long. **B** An example realisation of the mathematical model. In the right-hand plots green and magenta are used to differentiate two subsets of the initial patches ($t=0$, top) and their subsequent development ($t=25$, bottom). Black areas indicate the computational domain, $\varOmega$. The plot of initial condition is a blow-up of the centre of the whole domain. The scalebars represent 7 nondimensional space units. **C** Schematic of model initial condition. Initial populations (filled coloured circles) are placed in ${\varOmega }_{0}$, a small subdomain of the whole computational domain $\varOmega$ (both centred at the origin $O$).

Our mathematical model was motivated by experimental assays used to establish colony biofilms where the founding inoculum is placed on the surface of solidified nutrient agar. Within the inoculum footprint, individual (or small clusters of) bacteria settle at random locations and grow over time into a mature structured macroscale community (Fig. 1A). In the mathematical model, all the founding cells are assumed to have identical properties. However, to track the dynamics of biofilm growth we divided the founding cells into two groups, denoted by ${B}_{1}$ (shown in magenta) and ${B}_{2}$ (shown in green) (Fig. 1B). Note that we refer to ${B}_{1}$ and ${B}_{2}$ as *strains* for brevity, even though they represent two isogenic cell lineages that express different fluorescent proteins in a single-strain biofilm (Fig. 1A). In our theoretical framework, biofilm dynamics were reduced to the fundamental processes of *local growth* and *spatial spread* (more details below), which provided a species-independent representation of dual-strain biofilm growth. Suitably nondimensionalised (see Section S3), the model is given by

$$\frac{\partial {B}_{1}}{\partial t}=\nabla \cdot \left({Id}\left(1-\left({B}_{1}+{B}_{2}\right)\right){\nabla B}_{1}\right)+{B}_{1}\left(1-\left({B}_{1}+{B}_{2}\right)\right),$$

1 $$\frac{\partial {B}_{2}}{\partial t}=\nabla \cdot \left({Id}\left(1-\left({B}_{1}+{B}_{2}\right)\right)\nabla {B}_{2}\right)+{B}_{2}\left(1-({B}_{1}+{B}_{2})\right),$$

where, the variables ${0\le B}_{1}\left({{{{{\boldsymbol{x}}}}}},t\right),{B}_{2}\left({{{{{\boldsymbol{x}}}}}},t\right)\le 1$ denote the scaled densities of each strain, respectively at time $t\, > \,0$ (one nondimensional time unit corresponding to approx. 2.9 h) and at spatial position ${{{{{\boldsymbol{x}}}}}}\in \Omega$ (one nondimensional space unit corresponding to approx. 0.15 mm). The spatial domain $\Omega =\{{{{{{\boldsymbol{x}}}}}}\in {{\mathbb{R}}}^{2}:{||}{{{{{\boldsymbol{x}}}}}}{||}\le R\}$ is a two-dimensional disk, representing the biofilm growth medium (Fig. 1C). This simplification provided a significant reduction in computational cost and was motivated by an analysis of a previously published data set, in which we found a two-order of magnitude difference between biofilm diameter and biofilm thickness in *B. subtilis* NCIB 3610 [27]. The model is therefore unable to explicitly resolve density distributions along the vertical axis, for example, layering of subpopulation caused by gradients in environmental conditions [28–30] or topographical features such as ‘wrinkles’ [31]. However, it is fully capable of capturing overlap between subpopulations that are below the environmental carrying capacity and thus can track spatio-temporal coexistence. Moreover, as we show below, we find strong agreement between data obtained from two-dimensional in silico biofilms and data gathered from laboratory grown biofilms, which further supports the model simplification.

The initial conditions of the theoretical framework were motivated by the random positions at which bacteria settle on the agar within the inoculum footprint (Fig. 1A). In our theoretical framework, we represented the experimental inoculum footprint by a small disk ${\Omega }_{0}=\left\{{{{{{\boldsymbol{x}}}}}}\in \Omega :{||}{{{{{\boldsymbol{x}}}}}}{||} \; < \; {R}_{0}\right\}$ in the centre of the computational domain (Fig. 1C). We modelled the random deposition of bacteria by randomly placing ‘microcolonies’ within ${\Omega }_{0}$ at nodes of a triangulated spatial mesh of linear geometric order, used in the application of a finite element method to numerically solve the model equations (Fig. 1B, C). Each initial microcolony was assumed to only contain one strain and to be at carrying capacity (i.e., ${B}_{1}=1$ or ${B}_{2}=1$ within each microcolony). Unless otherwise stated, we used an even number ($N$) of initial microcolonies and assigned exactly $N/2$ to each strain at random. At spatial locations other than the assigned microcolonies, both densities were set to zero.

The size of a spatial mesh element used in the model (approx. $0.008{m}{m}^{2}$ in experimental parameters) was much larger than that of a single bacterial cell. This means that the initial conditions represented the experimental assays shortly after inoculation (typically after 24 h of incubation), at which time each bacterium (or small cluster of bacteria) had formed a *distinct, spatially separated microcolony*. Hence, the number of in silico *microcolonies*, $N,$ represented the number of bacteria used in the initial inoculum. Resolving the initial data at this spatial scale allowed analysis for founder densities $0\le N\le 824$. Using a selected set of values from that range was sufficient to capture clear trends (see below). The range covers biologically relevant founder densities, which generate mature colony biofilms with broadly similar morphologies (Supplementary Fig. S1). Additionally, to verify whether the observed trends could be extrapolated to $N \; > \; 824$, we represented high founder densities by piecewise spatially homogeneous initial conditions ${B}_{1}={B}_{2}=0.5$ in ${\Omega }_{0}$ and ${B}_{1}={B}_{2}=0$ otherwise.

The strains were assumed to grow logistically, with growth being limited by the total population, which could not exceed unity (after nondimensionalisation). Moreover, spatial propagation was described by diffusion as is common [32]. However, in our model, we employed a diffusion coefficient that decreased with increasing population size. This density dependence prevented merging of initially separated founding patches in the model and was invoked to capture experimental observations that indicated such colonies abut rather than merge on meeting [33, 34]. The indicator function ${Id}=1$ if ${B}_{1}+{B}_{2}\le 1$ and ${Id}=0$ otherwise guaranteed nonnegativity of the diffusion coefficients; this constrained the model to the physically relevant case and moreover ensured numerical stability during simulation.

Finally, we defined the *competitive outcome* score (for ${B}_{1}$) of the interaction to be the relative mass of strain ${B}_{1}$ i.e., ${B}_{1}^{\Omega }/({B}_{1}^{\Omega }+{B}_{2}^{\Omega })$ at the chosen end point ($t=T$) of our model simulation, where

$${B}_{i}^{\Omega }:={\int }_{\Omega }{B}_{i}({{{{{\boldsymbol{x}}}}}},T){{{{{\rm{d}}}}}}{{{{{\boldsymbol{x}}}}}},\,i=1,2.$$

The competitive outcome score lies in the interval $\left[{{{{\mathrm{0,1}}}}}\right]$ with the value 0.5 signifying a 1:1 ratio between the strains. Note that we could swap the indices without loss of generality to equivalently define the competitive outcome to be the relative mass of strain$\,{B}_{2}$ at the chosen end point.

### Low founder densities yield large variability in competitive outcomes

In the absence of spatial dynamics, the mathematical model predicted that the ratio between both strains would always remain constant $\left(\frac{d}{{dt}}\big(\frac{{B}_{1}}{{B}_{2}}\big)=0\right)$ and therefore that the competitive outcome would be determined by the initial ratio. To test whether such a relationship continued to hold in the full, spatially extended system, we examined data from simulations over a test range of initial founding cell densities. The initial strain ratio was selected to be 1:1 for each test.

**Fig. 2:**
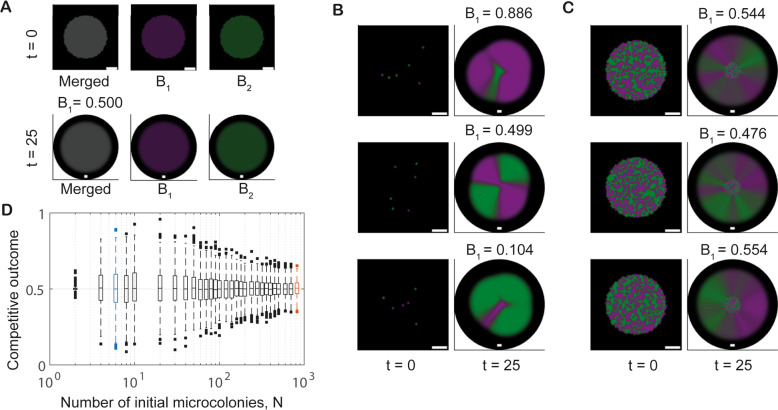
Spatial structure and variability in competitive outcome depend on founder density. **A**–**C** Example model realisations for different founder densities. All plots show the system’s initial conditions ($t=0$) and the outcomes after 25 time units. Plots visualising the systems’ states at $t=0$ show a blow-up of the subdomain ${\varOmega }_{0}$; plots visualising outcomes at $t=25$ show the full computational domain $\varOmega$ (black background). The scalebars are seven unit lengths long. **A** The outcome of simulations initialised with piecewise spatially homogeneous populations representing high founder density. The ‘Merged’ image channel shows both strains (grey colour corresponds to overlap); the ${B}_{1}$(green) and ${B}_{2}$ (magenta) channels only show single strain filters of the plot. **B** The range of outcomes observed for low founder density (number of initial cell patches ${N}=6$). **C** The range of outcomes for intermediate founder densities ($N=824$). In (**B**, **C**) only the ‘Merged’ channel is shown. **D** Variability in competitive outcome increases with decreasing founder density. Each boxplot contains data from 1000 model realisations. Blue and red boxplots correspond to the founder densities in **B** and **C**, respectively.

Model simulations using homogeneous initial conditions (representing high founder densities) consistently resulted in a competitive outcome score of 0.5 (i.e., strains in 1:1 ratio) with the strains remaining homogeneously distributed in space across the colony (Fig. 2A, Supplementary Movie S1). By contrast, independent model realisations using a specified number of microcolonies placed at randomly chosen locations representing low (${N}=6$) and intermediate (${N}=824$) founder densities, revealed significant variation in competitive outcome (Fig. 2B, C, Supplementary Movies S2 and S3). To explore this observed variability in more detail, we employed a Monte Carlo approach. For each fixed founder density $N$ within the selected set, 1000 independent model realisations were conducted. Data from these simulations revealed that the competitive outcome score for each founder density was normally distributed with mean 0.5. The standard deviation was relatively large for low founder densities ($N={{{{\mathrm{4,6,8,10}}}}}$) and decreased with further increases in $N$ (Fig. 2D). (Note the small standard deviation for $N=2$; see supplementary information for a discussion of this special case). Finally, our model predicted significant changes in the spatial organisation of the two strains within the biofilm in response to changing founder density, consistent with previous studies [14]. For high founder densities, isogenic *in silico* strains were predicted to coexist homogenously (Fig. 2A). However, as the founder density was decreased (decreasing $N$), homogeneous coexistence was gradually replaced by the formation of spatial sectors dominated by one strain or the other. Full segregation occurred for low founder densities (Fig. 2B, C).

### Access to free space determines competitive outcome

Next, we attempted to uncover the mechanism(s) by which low founder densities drive variability in competitive outcome. Motivated by [14], we first tested whether the initial separation between initial microcolonies of different types was the simple determinant. We did not find this to be the case for isogenic strain pairings in the mathematical model (Supplementary Fig. S2).

As an alternative, we hypothesised that a microcolony surrounded by others may have little impact on competitive outcome as its contribution to biofilm growth would be ultimately limited. On the other hand, microcolonies located close to the boundary of the biofilm inoculum would be free to expand radially and thus could make a more significant contribution to the competitive outcome (for an example timelapse video see Movie S3). Hence, we explored whether competitive outcome was correlated to a strain’s *potential for radial expansion* beyond the inoculum. To do so, we assumed the potential for radial expansion to be solely determined by the geographical locations of a strain’s initial microcolonies. We then defined an appropriate score for this potential as follows. First, a circle was drawn that enclosed the initial microcolonies. Second, each point on the circle was associated with the nearest microcolony and assigned to that strain. Third, the total arc length on the circle associated with each strain was computed. Finally, the *access to free space score* (AFS score) for strain ${B}_{1}$, denoted AFS_1_, was then computed as the ratio of the total arc length associated with ${B}_{1}$ to the circumference of the circle. Therefore, $0\le {{{{{\rm{AF}}}}}}{{{{{{\rm{S}}}}}}}_{1}\le 1$ quantified strain ${B}_{1}$’s hypothesised potential to contribute to radial biofilm expansion. It is straightforward to confirm that the AFS score for strain ${B}_{2}$, ${{{{{\rm{AF}}}}}}{{{{{{\rm{S}}}}}}}_{2}=1-{{{{{\rm{AF}}}}}}{{{{{{\rm{S}}}}}}}_{1}$. See Section S4.2 and Supplementary Figs. S3 and S4 for a mathematically rigorous definition of the AFS score.

**Fig. 3:**
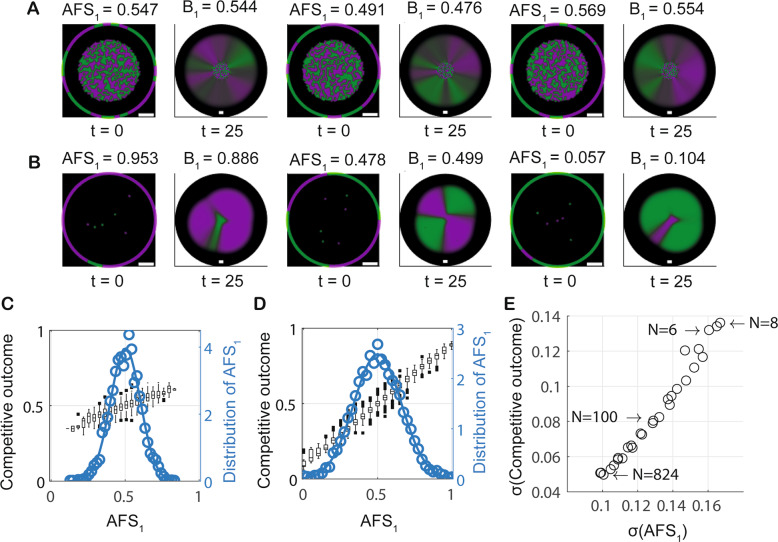
Access to free space determines competitive outcome. **A**, **B** Example model realisations for different founder densities. All plots show system initial conditions ($t=0$) with the reference circle used to compute the AFS score (the circle is rescaled for visualisation purposes) and outcomes after 25 time units. The founder densities are $N=824$ and $N=6$ in **A** and **B**, respectively. Plots visualising system states at $t=0$ show a blow-up of the subdomain ${\varOmega }_{0}$; plots visualising outcomes at $t=25$ show the full computational domain $\varOmega$ (black background). The scalebars are seven unit lengths long. **C**, **D** The relation between the AFS score ${AF}{S}_{1}$, and competitive outcome is shown for intermediate founder density ($N=824$) and low founder density ($N=6$) in **C** and **D**, respectively. Data were obtained from 5000 model realisations and cover the continuum of ${AF}{S}_{1}$. The observed probability density function for AFS is shown (circular markers); along with the density function of a fitted normal distribution ($\mu \approx 0.5,\sigma \approx 0.10$ in **C**, $\mu \approx 0.5,\sigma \approx 0.16$ in **D**) (solid line). **E** The relation between the standard deviations of the AFS score ${AF}{S}_{1}$ and the competitive outcome. Each data point (circle) represents a different founder density and contains information from 1000 model realisations.

We explored the utility of the AFS score using $N=6$ and $N=824$ as representatives of low and intermediate founder cell densities, respectively. We increased the number of model realisations to 5000 for each of the selected values of *N* to ensure improved accuracy of our data analysis. The AFS score was then calculated for each of the 10,000 initial conditions (see examples Fig. 3A, B). On completion of each simulation, the corresponding competitive outcome score was computed. Analysis of these model data confirmed that the AFS score accurately predicts competitive outcome: for each fixed founder density, the AFS score unfolds the variation shown in Fig. 2D, yielding a positive, linear relationship between AFS_1_ and competitive outcome for ${B}_{1}$ (Fig. 3C, D). For each of the selected values of $N$, initial configurations of microcolonies with a low AFS_1_ score predictably generated a low competitive outcome for ${B}_{1}$. Correspondingly, initial configurations with a high AFS_1_ score predictably generated a high competitive outcome for ${B}_{1}$. The slope of this linear relationship provided a deterministic quantification of the variability of competitive outcomes for a given founder density (cf. Fig. 3C, D, Supplemental text).

We subsequently established that the predictive power of the AFS score was maintained across the range of founder densities considered in the model. Additionally, the variation in the AFS score was shown to decrease with increasing founder density (cf. Fig. 3C, D). Further, we revealed strong correlation between variation in AFS score and variation in competitive outcome (Fig. 3E). Therefore, for increasing founder density, the observed decrease in variation in competitive outcome can be directly attributed to the decrease in variation in the AFS score.

### Dual strain single-isolate biofilm assays confirm modelling hypotheses

**Fig. 4:**
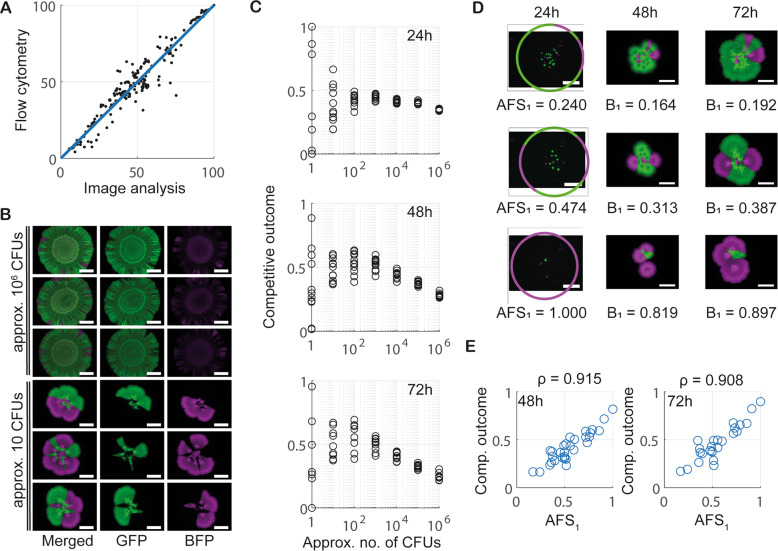
Experimental data confirm modelling hypotheses. **A** Comparison of image analysis with flow cytometry. A scatter plot comparing measurements of relative density of the mTagBFP-labelled strain obtained from image analysis and flow cytometry is shown. Each data point corresponds to one biofilm, which was imaged before being analysed by flow cytometry. The data contains measurements taken from all strain pairs, all founder densities, and all time points. The solid blue line shows the identity $x=y$, with the coefficient of determination being ${R}^{2}=0.91$. **B** Example images of single-strain biofilms consisting of GFP (green$,{B}_{1}$) and mTagBFP (magenta, ${B}_{2}$) labelled copies of 3610. Taken after 72 h of incubation and shown for two different founder densities (scalebar 5 mm). **C** Strain density data. Competitive outcome measurements taken after 24 h, 48 h and 72 h of biofilm incubation. Plotted are technical repeats from one biological repeat of the experiment. The full data set is presented in Fig. S5A. **D** Example visualisations of AFS score calculations. Three example biofilms images at 24 h (left), 48 h (middle) and 72 h (right). The strains are as described in **B**. Images at 24 h show the reference circle used for the AFS_1_ score. **E** The relationship between AFS_1_ and competitive outcome for ${B}_{1}$. AFS was calculated from images taken at 24 h, and competitive outcome for ${B}_{1}$ after 48 h (left, $n=30$) and 72 h (right, $n=25$). The linear correlation coefficient $\rho$ is indicated.

Next, we aimed to test the hypotheses put forward by the mathematical model. We selected an isogenic pair of *Bacillus subtilis* strains derived from isolate NCIB 3610 that constitutively produced the green fluorescent protein GFP (NRS6942, shown in green, Table S1) and the blue fluorescent protein mTagBFP (NRS6932, shown in magenta, Tables S1 and S2), respectively. In line with the modelling assumption, the isolates were mixed in a 1:1 ratio at a defined initial cell density (we used an OD_600_ of 1) and this cell culture was serially diluted prior to inoculating the colony biofilms (Section S7). Thus, biofilms were inoculated using ~10^6^ CFUs and dilutions in 10-fold increments to order 1 CFU. For each founder density, 12 technical replicates were performed to provide a meaningful sample size, and the experiment was repeated on three independent occasions. We used a non-destructive colony biofilm image analysis approach, to measure the relative mass (and hence the competitive outcome) of the two isogenic strains at 24 h, 48 h, 72 h after inoculation (see Section S10). We confirmed that the output from the image analysis correlated well with data generated by disruption of the colony biofilm and analysis of the relative strain proportions determined using single cells analysis by flow cytometry (Fig. 4A) (see also [35]). The mTagBFP labelled strain consistently performed marginally worse than the GFP labelled competitor at high founder densities in co-culture, which suggests some impact on competitive fitness (Fig. 4B, C). To allow comparison with results from the mathematical model, we denoted the mTagBFP (NRS6932, shown in magenta) and GFP (NRS6942, shown in green) strains as ${B}_{1}$ and ${B}_{2}$, respectively, with associate AFS scores AFS_1_ and AFS_2_

Our experimental analysis proved consistent with the model predictions. High founder densities resulted in a broadly homogenous distribution of both strains over the footprint of the biofilm, while low founder densities led to a high degree of spatial segregation of the strains within the mature biofilm (Fig. 4B, see also [14]). Additionally, analysis of experimental data confirmed that variability in competitive outcome increased with decreasing founder density (Fig. 4B, C, Supplementary Fig. S5A). For founder densities equivalent to $\sim$10^3^ to $\sim$10^6^ CFUs, the competitive outcome was consistent across each set of technical replicates. By contrast, for founder densities equivalent to $\sim$1 to $\sim$10^2^ CFUs, the competitive outcome was variable across each set of technical replicates. We noted that variability in competitive outcome, at all initial founder densities, was marginally amplified over time.

We assumed the process of repeated dilution and selection of the inoculum volume may not guarantee an exact cell count and/or initial strain ratio of 1:1 at lower founder densities. Indeed, for low founder densities after 24 hrs incubation, we observed inconsistencies in the number and ratio of CFUs deposited (Supplementary Fig. S5B). We therefore considered whether these inconsistencies in the biofilm inocula contributed to the observed variability in competitive outcome. To explore this in more detail, we first implemented a combinatorial ‘cell picking’ model that mathematically simulated the process of selecting the small inoculum volume from a larger cell culture (see Section S4.3). This process identified a threshold of ${\sim} {10}^{2}$ CFUs below which variability in cell number and/or strain ratio could measurably deviate from their intended values in our experimental assay. Above this threshold, the combinatorial argument predicted limited deviation from the intended values (Supplementary Fig. S6A). Coupling these theoretical predictions with our experimental observations (Supplementary Fig. S5B), we concluded that any observed variability in competitive outcome cannot be a consequence of a measurable deviation in the inoculum composition for colony biofilms founded with $\sim {10}^{2}$ CFUs or higher.

We next wanted to determine whether the predictive power of the AFS score could be used to connect experimental initial configurations of the bacteria with the observed competitive outcome. To do this accurately, we required that the founding bacteria remained spatially separated as small colonies until an image was taken at 24 h (the earliest imaging time-point, see Fig. 4D). Therefore, we only used founder densities lower than 10^2^ CFUs. However, the above noted inconsistencies in initial strain ratios and cell counts at these densities raised the question of whether AFS could still accurately predict competitive outcome. To test this, we repeated our Monte Carlo simulations of (1) in which the number of initial microcolonies for each strain was drawn using the combinatorial cell picking model, rather than being a fixed number and in a 1:1 ratio. Analysing the resulting simulation data for model (1) confirmed that the predictive power of the AFS score was robust to any ‘naturally-occurring’ variation in the initial strain ratio (Supplementary Fig. S6B). Correspondingly, our analysis of the experimental data revealed a strong correlation between a strain’s AFS score and the competitive outcome measured at 48 h and 72 h after incubation (Fig. 4E).

### A modelling framework for non-isogenic strains

We have established that for isogenic strains, the initial configuration of founding bacteria determines the competitive outcome in a ‘race for space’ and that the AFS score can accurately predict which strain will dominate. A natural question that follows is what would happen if this race for space was influenced by antagonistic interactions such as killing or growth inhibition. Therefore, we considered the effect of introducing a local (e.g., contact-dependent or short-range non-contact dependent) antagonistic mechanism that causes a reduction in strain net growth. In an extension of our theoretical framework (1), constants describing the ratios between the strains’ maximum growth rates in the absence of competition ($r$), diffusion coefficients ($d$) and competition coefficients ($c$) were introduced to allow for the possibility of differences in strain properties. This resulted in the following system obtained after a suitable nondimensionalisation (see Section S3):

$$\frac{\partial {B}_{1}}{\partial t}=\nabla \cdot \left({Id}\left(1-\frac{{B}_{1}+{B}_{2}}{k}\right){\nabla B}_{1}\right)+{B}_{1}\left(1-\frac{{B}_{1}+{B}_{2}}{k}\right)-{B}_{1}{B}_{2},$$

2 $$\frac{\partial {B}_{2}}{\partial t}=\nabla \cdot \left({Id}\cdot d\left(1-\frac{{B}_{1}+{B}_{2}}{k}\right)\nabla {B}_{2}\right)+{{rB}}_{2}\left(1-\frac{{B}_{1}+{B}_{2}}{k}\right)-c{B}_{1}{B}_{2}.$$

Here, the indicator function ${Id}=1$ if ${B}_{1}+{B}_{2}\le k$ and ${Id}=0$ otherwise, where *k* is the nondimensional carrying capacity. To start, strains were assumed to possess identical growth dynamics in the absence of competitors (i.e., *r* $=1,{d}=1$), but to significantly differ in their ability to negatively impact the competitor strain. For the simulations we set $c=0.2$ representing a five-fold difference in competition strength, with ${B}_{2}$ being the more effective competitor. A linear stability analysis of model [4] confirmed that in this case and for a homogeneous initial distribution of the strains in a 1:1 ratio, ${B}_{2}$ wins the interaction. For this reason, we therefore refer to ${B}_{2}$ as the (intrinsically) *stronger strain* and to ${B}_{1}$ as the (intrinsically) *weaker strain* in the following.

The assumption of identical growth dynamics allowed us to focus on the impact of antagonistic interactions on competitive outcome. We anticipated that this assumption was unlikely to hold for non-isogenic strains in experimental settings and therefore we examined (as will be discussed later) the impact of changes to the parameters $r,{d}$ and $c$. Subsequently, we showed the effect of such parameter variation to be limited.

### Spatial segregation induced by low founder densities enables coexistence

**Fig. 5:**
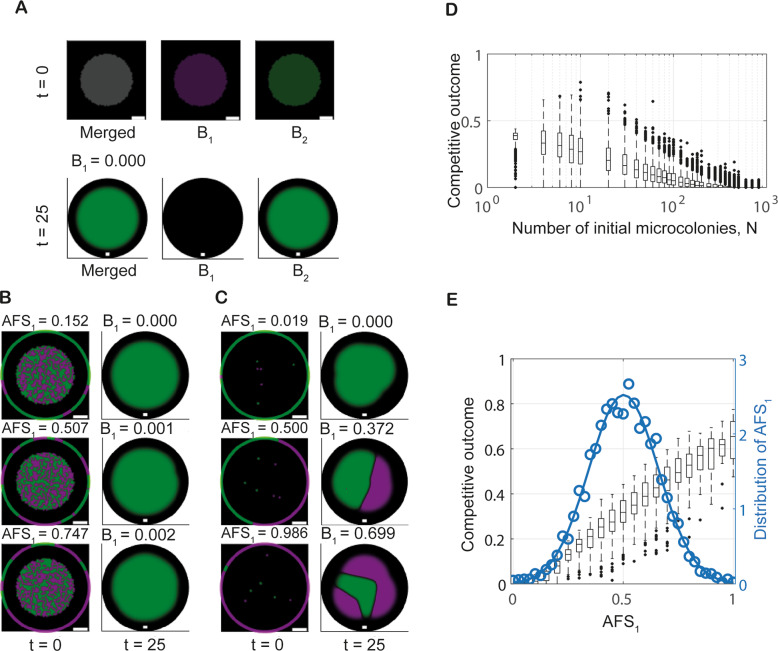
Modelling data for a non-isogenic strain pair with local antagonistic interactions. **A**–**C** Example model realisations for high (**A**), intermediate (**B**) and low (**C**) founder density are shown. **A** the Merged image channel shows both strains (grey colour corresponds to overlap), the ${B}_{1}$ and ${B}_{2}$ channels only show single strain filters of the plot. In **B**, **C** only the Merged channel is shown. Plots visualising system states at $t=0$ show a blow-up of the subdomain ${\varOmega }_{0}$ and the circles used to calculate the AFS scores around the initial conditions are not to scale. Plots visualising outcomes at $t=25$ show the full computational domain $\varOmega$ (black background). The scalebars are seven unit lengths long. **D** The relation between founder density and competitive outcome. Each boxplot contains data from 1000 model realisations. **E** The relation between the AFS score ${AF}{S}_{1}$, and competitive outcome for one fixed founder density ($N=6$). Data were obtained from 5000 model realisations and covers the continuum of ${AF}{S}_{1}$. The observed probability density function for AFS is shown (circular markers); the density function of a fitted normal distribution ($\mu \approx 0.5,\sigma \approx 0.16$) as a solid line.

In the context of local antagonistic interactions, low founder densities were expected to offer protection for the weaker strain by driving spatial segregation and the formation of enclaves. Test simulations supported this hypothesis. Model realisations with high (spatially uniform initial conditions) and intermediate ($N=824$) founder densities consistently led to competitive exclusion of the weaker strain (Fig. 5A, B, Supplementary Movies S4 and S5), while model realisations with low founder densities ($N=6$) resulted in coexistence with the strains being spatially segregated (Fig. 5C). Once established during early stages of the model simulation, spatial segregation was conserved. However, the stronger strain continually invaded its competitor’s clusters along strain-to-strain interfaces and eventually took over the biofilm centre. Simultaneously, the weaker strain enlarged its sectors due to unimpeded growth on the biofilm edge. Coexistence, as measured by competitive outcome was achieved by a balance of these processes (Supplementary Movie S6).

Low founder densities generated significant variation in competitive outcome (Fig. 5C). In particular, outcomes were observed for which the weaker strain ${B}_{1}$ coexisted with, *and could even outperform*, the stronger strain ${B}_{2}$. To better understand the impact of founder density, we performed Monte Carlo simulations with 1000 independent model realisations for each founder density $N$ in our test range. Data from these simulations revealed both the mean and variation of competitive outcome for the weaker strain increased with decreasing founder density (Fig. 5D).

### Access to free space determines competitive outcome for low founder densities

The mathematical model consistently predicted competitive exclusion of the weaker strain at intermediate and high founder densities (Fig. 5A, B). Hence, in these cases, the AFS score no longer provided a meaningful predictor of competitive outcome. Rather, the model predicted the outcome to be dominated by the local antagonisms. However, as detailed above, low founder densities ($N$ = 6) resulted in a highly variable competitive outcome and therefore we explored whether the AFS score remained an accurate predictor in this case. The simulation data confirmed that for this fixed number $N$, the AFS score remained capable of accurately unfolding the observed variation in competitive outcome (Fig. 5E). Thus, initial strain configurations with a low AFS_1_ predictably generated a low competitive outcome for ${B}_{1}$. The reciprocal was also maintained where initial strain configurations with high AFS_1_ predictably generated high competitive outcome for ${B}_{1}$. As for isogenic strains, this relationship was found to be linear with the slope providing a measure of the deterministic range of competitive outcomes for a given founder density. The relationship between AFS and competitive outcome was again shown to be robust to natural variation in the initial strain ratio inherent in low founding cell densities (Supplementary Fig. S6C).

Our mathematical model predicted that coexistence remained possible over a range of maximum growth rates, $r$ (within a two-fold difference between dimensional strain growth rates in the absence of competition), diffusion coefficients, $d$ (within a three-fold difference between dimensional diffusion coefficients), and most surprisingly, *any* values of the competition coefficient, $c$ (Section S6 and Supplementary Fig. S7A–C). In particular, we showed that a strain required extreme competition efficiency ($c$ very large) in order to compensate for being slower in growth ($d,r \; > \; 1$) (Supplementary Fig. S7D). Finally, the predictive power of the AFS score was preserved over the parameter range tested (Supplementary Fig. S7E, F).

### Dual-isolate biofilm assays - selection of a competition partner

**Fig. 6:**
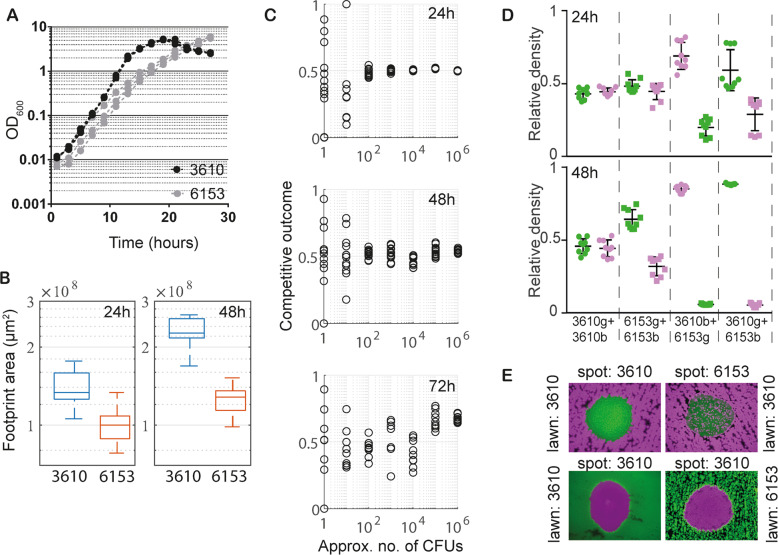
Selection of a competitive strain. **A** Growth curves of 3610 (black) and 6153 (grey) in MSgg cultures at 30 °C. The three lines shown for each isolate represent separate biological repeats. **B** Biofilm footprint area of single-strain 3610 and 6153 biofilms. Data from 18 and 16 biofilms are shown for the 24 h and 48 h timepoint, respectively. **C** Competitive outcome data from colony biofilm assays of isogenic 6153 biofilms are shown after 24 h, 48 h and 72 h of incubation. Plotted are the technical repeats from one biological repeat. The full data set is presented in Supplementary Fig. S8A. **D** Flow cytometry data of mixed biofilms grown for 24, 48, and 72 h at 30 °C on MSgg media. Isolate names followed by ‘g’ represent strains constitutively producing  GFP, (green on the graph). Isolate names followed by ‘b’ indicate strains constitutively producing mTagBFP, (magenta on the graph). Three biological and three technical replicates were performed for each strain mix and timepoint and all data points are shown. The error bars represent the mean standard deviation. **E** Halo formation assays on MSgg agar plates at 24 h of growth. Strains producing mTagBFP (magenta) and GFP (green) are shown.

To experimentally test our model predictions, we needed to identify a suitable partner for NCIB 3610. We chose a *Bacillus subtilis* strain called NRS6153 (hereafter 6153). This selection was made because (i) 6153 is a genetically competent wild type strain with no known auxotrophies [36]); (ii) in liquid culture conditions the generation times of the two strains are within ~1.5-fold of each other (Fig. 6A); (iii) under biofilm conditions, single strain biofilms of both isolates have footprint sizes that are within $\sim$2-fold of each other (Fig. 6B); (iv) across a broad range of founder densities, the competitive outcome of an isogenic pairing of 6153 isolates in a colony biofilm is broadly similar to that of an isogenic pairing of 3610 strains, albeit with more variability in the competitive outcome at the 72-h time point for high founder densities (cf. Fig. 4C (Supplementary Fig. S5A) and Fig. 6C (Supplementary Fig. S8A)); (v) when a colony biofilm is founded at high density with marked strains of 3610 and 6153 starting at an initial 1:1 ratio, 6153 is consistently outcompeted by 3610 (and hence defines 3610 as the *stronger strain* in the context of this study) (Fig. 6D); and (vi) using an antibiosis halo formation assay, interrogation of the interaction between 3610 and 6153 showed no evidence of contact-independent growth inhibition (Fig. 6E). In combination, these data allow us to infer that the mode of competition during co-culture in the colony biofilm is locally antagonistic.

### Dual-isolate biofilm assays confirm modelling hypotheses

**Fig. 7:**
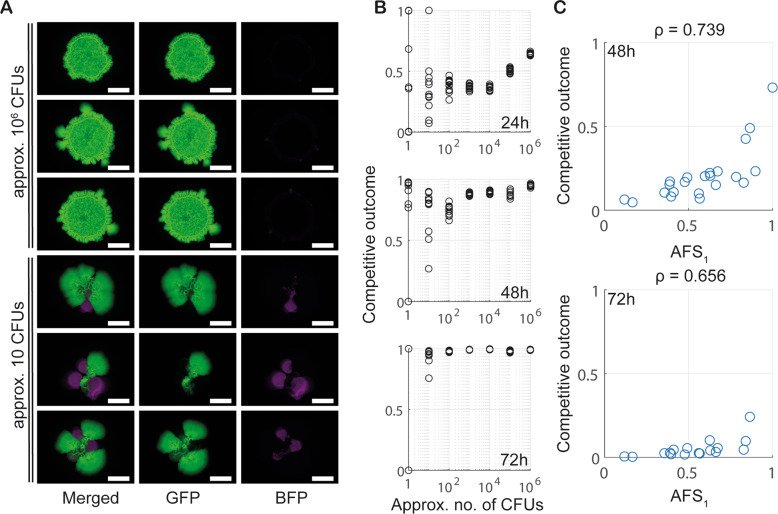
Experimental data for a non-isogenic strain pair with local antagonistic interactions. **A** Example dual-strain biofilms (3610 labelled with GFP (green), 6153 labelled with mTagBFP (magenta)). Images taken after 72 h of incubation for two different founder densities. Scalebars as in Fig. 2. **B** Competitive outcome data for 3610 in the 3610/6153 pair after 24 h, 48 h and 72 h of biofilm incubation. Plotted are technical repeats from one biological repeat of the experiment. The full data set is presented in Supplementary Fig. S8B. **C** The relationship between AFS and competitive outcome for 6153. AFS_1_ was calculated based on images taken after 24 h of biofilm incubation, and competitive outcome after 48 h (top, ${n}=22$) and 72 h (bottom, $n=17$).

We performed dual strain biofilm assays competing 3610 and 6153 over a wide range of founder densities. These competitive assays confirmed the modelling prediction that in biofilms inoculated at low founder densities, coexistence within a non-isogenic strain pair is enabled by spatial segregation (Fig. 7A). Under such conditions, the intrinsically weaker strain (6153) formed spatial sectors and thus was able to coexist with the stronger strain (3610) through spatial segregation (Fig. 7A, B). In contrast, and again as predicted by the mathematical model (and reported during the selection of strain 6153 as a competition partner), for biofilms inoculated at high founder density, 3610 competitively excluded 6153 (Fig. 7A, B, Supplementary Fig. S8B). Finally, a computation of AFS scores based on images taken after 24 h of incubation showed strong correlation between a strain’s AFS score and its competitive outcome after both 48 h and 72 h of incubation for both 6153 alone and when in co-culture with 3610 (Supplementary Figs. S9 and 7C).

## Discussion

Significant advances in the understanding of competition dynamics within bacterial biofilms have been made in recent years [13, 14, 16–19, 26, 37]. However, many basic questions remain open, including how the initial configuration of the founding cells impacts the morphology and behaviour of the mature community. Here, using a multi-disciplinary approach, we have revealed that a *race-for-space* dominated competition dynamics in single- and dual-strain colony biofilms inoculated at low founder densities. This even occurred in cases where the local antagonistic mechanisms strongly favoured survival of one strain over the other. In short, we established that range expansion dominated local antagonisms in biofilms inoculated with low founder densities. We showed that the resulting spatial structure supported coexistence of strains and revealed that it could lead to the counter-intuitive outcome of the *weaker* strain outperforming the *stronger* in terms of competitive outcome (relative biomass). Moreover, we established that a particular measure of the initial configuration of founder cells reliably predicted the resulting spatial structure and the relative densities of the strains in the mature biofilm. Our predictive measure, which characterises a strain’s *access to free space*, enabled us to disentangle experimentally observed variability in structure and competitive outcome in low founder density biofilms revealing this to be a natural, and predictable consequence of competition for space. This predictor proved to be remarkably robust to changes in the strain properties and initial strain ratio.

Our experimental system focussed on a dual-strain *B. subtilis* biofilm assay in which strains compete for space and interact through local antagonistic mechanisms that reduce one strain’s net growth via contact-dependent killing or contact-dependent growth inhibition mechanisms, for example. However, we propose that due to the lack of species-specific assumptions in our mathematical model, our results could be extended to other microorganisms whose dynamics can be described by logistic growth, diffusion and antagonisms. As well as contact-dependent methods of competition, antagonisms could also include contact-independent actions, such as the secretion of diffusible antimicrobials [38]. It is known that certain toxins have a restricted ability to penetrate cells distal to their production site [39]. Hence, provided the spatial scale on which the antagonistic contact-independent interaction mechanism acts is sufficiently short, spatial segregation induced by the configuration of founder cells is still likely to drive dynamics very similar to those observed for contact-dependent mechanisms. Tests of this hypothesis could be the subject of future work and may be performed through an extension of our theoretical framework through the explicit description of toxin dynamics and an experimental approach using a different choice of strains. In this way, it may be possible to determine critical penetration depths at which the competitive dynamics alter significantly from those discussed here. By contrast, we do not expect our results to extend to cooperative interactions, such as cross feeding [40]. As cooperation benefits from spatial mixing rather than segregation, growth dynamics are not driven by competition for space. Therefore, predictions of biofilm spatial structure would require a different approach, which again could be explored through an extension of our model system.

Consistent with previous studies, we have shown that spatial segregation provides protection from local antagonisms and is induced by low founder densities [14, 16, 17, 21, 26]. Our findings highlight that the spatial structure favours strains that would be outcompeted in well-mixed contexts and allows them to persist in the more complex setting of colony biofilms. It is worth noting that low founder density is not the only mechanism that can induce spatial segregation of cell linages in a biofilm. Spatial structure can also be induced by genetic drift due to the small size of the population within the biofilm edge that contributes to radial expansion [13, 18, 19, 41]. However, spatial segregation via genetic drift is a gradual process that requires strains to coexist without spatial structure in the biofilm centre [13, 18, 19, 42]. It is thus unlikely to affect biofilm phenotype if antagonistic interactions that prevent coexistence in the biofilm centre dominate competitive interactions. Indeed, spatial structure induced by genetic drift is commonly associated with prevention of exploitation of co-operators by cheaters in social dynamics, rather than protection from antagonistic actions [13, 41, 43, 44].

The development of a deeper understanding of competition dynamics in multi-strain biofilms is an essential precursor for the optimal design and implementation of industrial applications. For example, biofilm-forming microbial species in the rhizosphere can mutualistically interact with plant roots and are therefore used as biofertilizers and biopesticides. Biofilms on plant roots can supply plants with fixed nitrogen and provide protection from plant pathogens [45–47] in exchange for root exudates [48, 49], such as carbon [50]. *B. subtilis* is a species widely used in biocontrol [46, 51, 52]. However, *B. subtilis* has a large and open pangenome [53, 54] and only select isolates have been shown to possess traits associated with successful applications as biocontrol agents [55]. A biocontrol agent can only be successfully applied if it manages to coexist (or outcompete) other strains already present [56]. These and many other examples, such as the applications of biofilms in wastewater treatment [6], microbial fuel cells [57] and corrosion prevention [58], illustrate the need to better understand interstrain interactions within biofilms growing in complex and potentially continually changing environments. Therefore, we argue that future work focusing on enhancing our understanding of spatial structure and competition within multi-strain biofilms will be critical to optimise our ability to maximise the positive impact of biofilms.

## Supplementary information

Supplementary Material

Movie S1

Movie S2

Movie S3

Movie S4

Movie S5

Movie S6

## Data Availability

Computational code is available on Github and has been archived by Zenodo [59]. The experimental datasets have been achieved using BioStudies [60].
