# Supplementary material for: Founder cell configuration drives competitive outcome within colony biofilms
Source: ISME J. 2022 Feb 5;16(6):1512–22. doi: 10.1038/s41396-022-01198-8 (PMC9122948; doi:10.1038/s41396-022-01198-8)
Supplement: Supplementary file 1 — Supplementary Material [file 41396_2022_1198_MOESM1_ESM.pdf]

Founder cell configuration drives competitive outcome within colony biofilms

Supplementary Material

Lukas Eigentler[1,2], Margarita Kalamara[1], Graeme Ball[3], Cait E. MacPhee[4], Nicola R. Stanley-Wall[1,*], Fordyce A. Davidson[2,*]

[1] Division of Molecular Microbiology, School of Life Sciences, University of Dundee, Dundee DD1 5EH, United Kingdom
[2] Division of Mathematics, School of Science and Engineering, University of Dundee, Dundee DD1 4HN, United Kingdom
[3] Dundee Imaging Facility, School of Life Sciences, University of Dundee, Dundee DD1 5HN, United Kingdom
[4] School of Physics and Astronomy, The University of Edinburgh, Edinburgh EH9 3FD, UK

*Joint Corresponding Authors: f.a.davidson@dundee.ac.uk; N.R.Stanleywall@dundee.ac.uk

Contents

S1 Set-up and supplementary figures 3
S2 Supplemental movies 12
S3 Models and nondimensionalisations 13
S4 Methods for model analysis 14
S4.1 Model implementation 14
S4.2 Definition of access to free space score 14
S4.3 Combinatorial cell picking model 15
S5 Notes on variability in competitive outcome 16
S6 Model hypotheses are robust to differences in strain growth dynamics 16
S7 Experimental methods 18
S7.1 Growth conditions 18
S7.2 Biofilm formation assays 18
S7.3 Determining cell counts 18
S7.4 Single cell analysis by flow cytometry 19
S7.5 Antibiosis halo assays 19
S7.6 Growth curves 19
S8 *Bacillus subtilis* strains 20
S8.1 Strain construction 20
S8.2 Strain table 20
S9 Sequence of *mTagBFP* coding region 22
S10 Image analysis 23
S10.1 Access to free space 23
S11 References 24

Table of figures

Figure S1**: The mature biofilm morphology is robust to the number of founding cells** 3
Figure S2: **Initial interstrain separation cannot predict competitive outcome in isogenic strain pairs** 4
Figure S3: **Schematic of the derivation of the access to free space score** 5
Figure S4: **The impact of $R \coloneqq nR_0$ on the access to free space score** 6
Figure S5: **Experimental data of all three biological replicates for isogenic strain pair** 7
Figure S6: **Data from combinatorial cell picking model.** 9
Figure S7: **Impact of changes to model parameters on competitive outcome. A-D** 9
Figure S8: **Experimental data of all three biological replicates for non-isogenic strain pair with local antagonistic interactions.** 10
Figure S9: **Access to free space as a predictor of competitive outcome for the isogenic 6153 pair** 11

S1 Set-up and supplementary figures

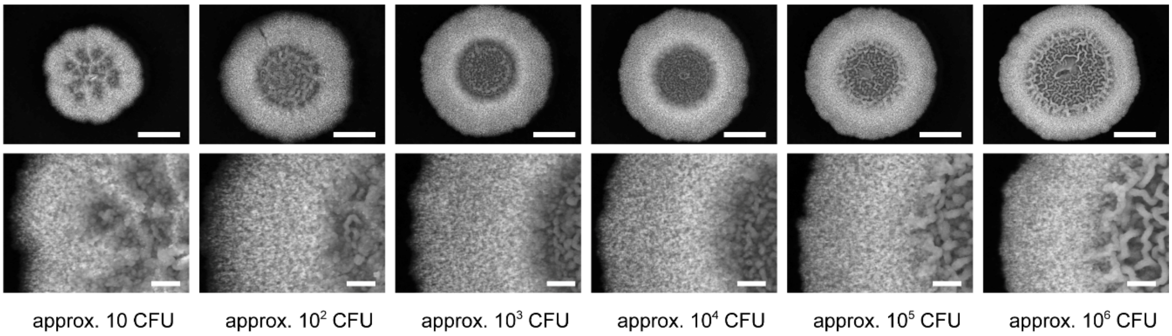

*Figure S1**: The mature biofilm morphology is robust to the number of founding cells.*** B. subtilis *strain NCIB 3610 was used to establish colony biofilms using a range of founder densities. The biofilms were grown at 30°C for 48 hours prior to photography. The scale bar represents 5 mm.*

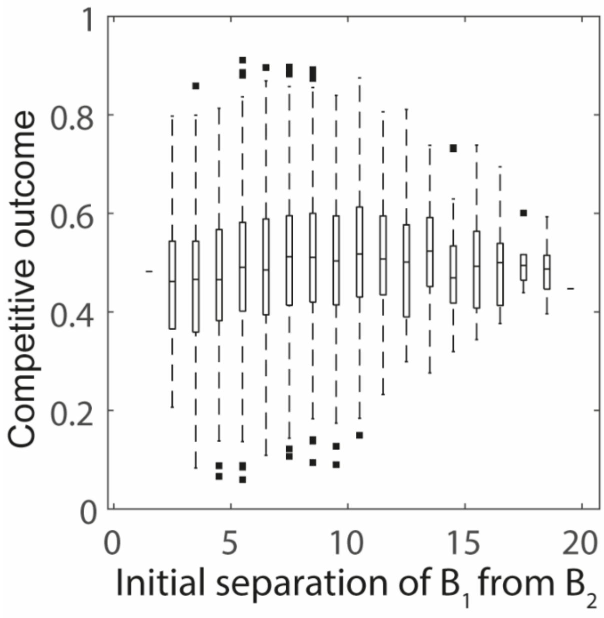

Figure S2: ***Initial interstrain separation cannot predict competitive outcome in isogenic strain pairs.*** *The mean initial separation of B_1 microcolonies to their nearest B_2 microcolony is plotted against the corresponding competitive outcome. The initial separation between the microcolonies was split into bins for visualisation purposes. No clear relation between the two quantities exists.*

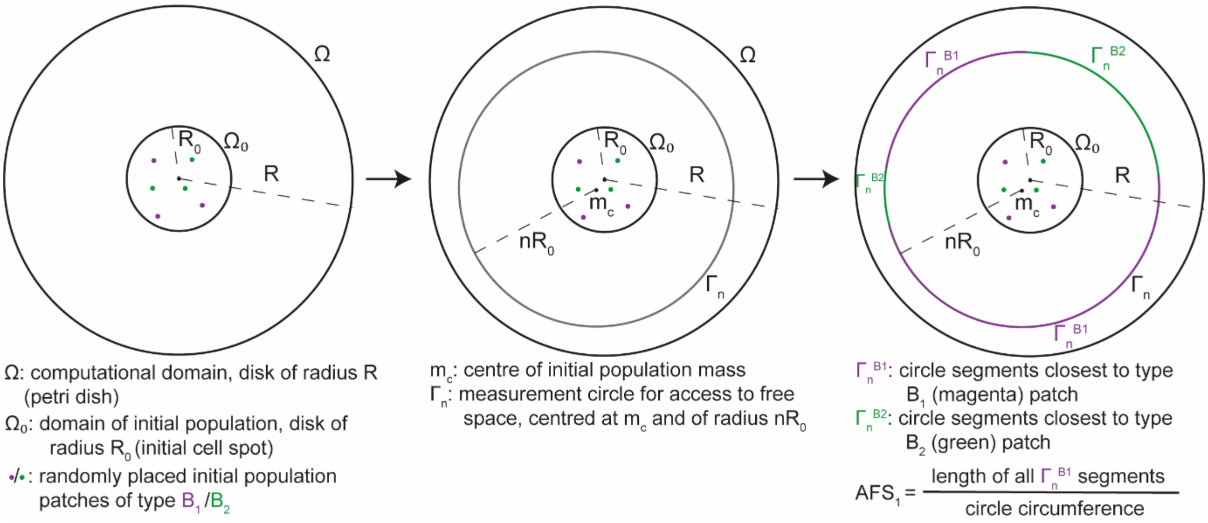

Figure S3: ***Schematic of the derivation of the access to free space score.*** *A reference circle Γ_n of radius nR_0 is drawn centred at m_c, which is the centre of mass of the initial population. Circle segments are assigned to set $\Gamma_n^{B_1}$if the closest strain patch belongs to B_1, and to $\Gamma_n^{B_2}$ otherwise. For ease of visualisation, these segments are given the same colour as the nearest patch. The colour of the circle changes at points where nearest patches of different strains are equidistant to the point on the circle. The ratio of the total length of the magenta segments to the circumference of Γ_n defines the access to free space score AFS_1 of strain B_1, and correspondingly for strain B_2 (green).*

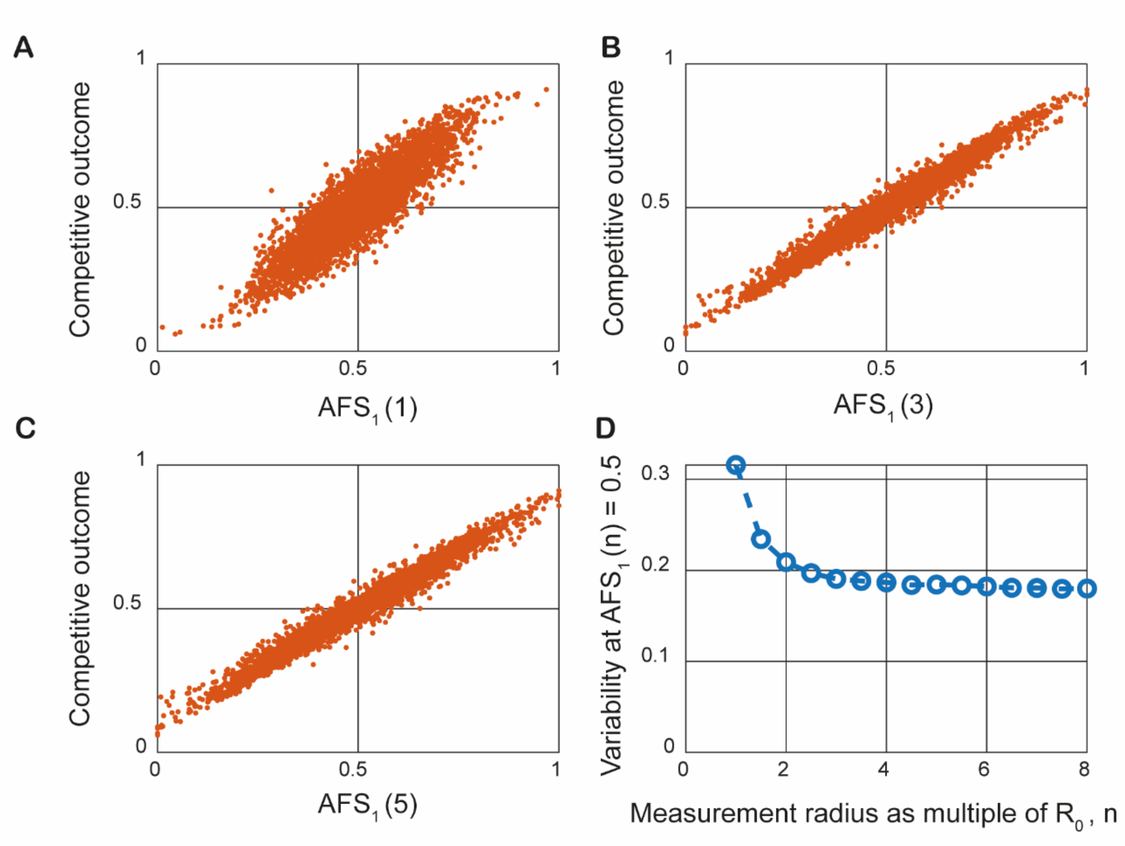

Figure S4: ***The impact of $R \coloneqq nR_0$ on the access to free space score. A-C*** *Scatter plots of outcome strain density B_1 against the access to free space score AFS_1 of the system's initial condition is shown for three different values of n.* ***D*** *Range of B_1 in which 95% of data with an access to free space score $AFS_1 \approx 0.5$ is contained. Significant changes to AFS_1 occur as n increases from its minimum $n = 1$, but for sufficiently large n, changes in n have negligible impact on AFS_1. Each data point represents one model realisations with $N = 6$ initial cell patches of two isogenic theoretical strains.*

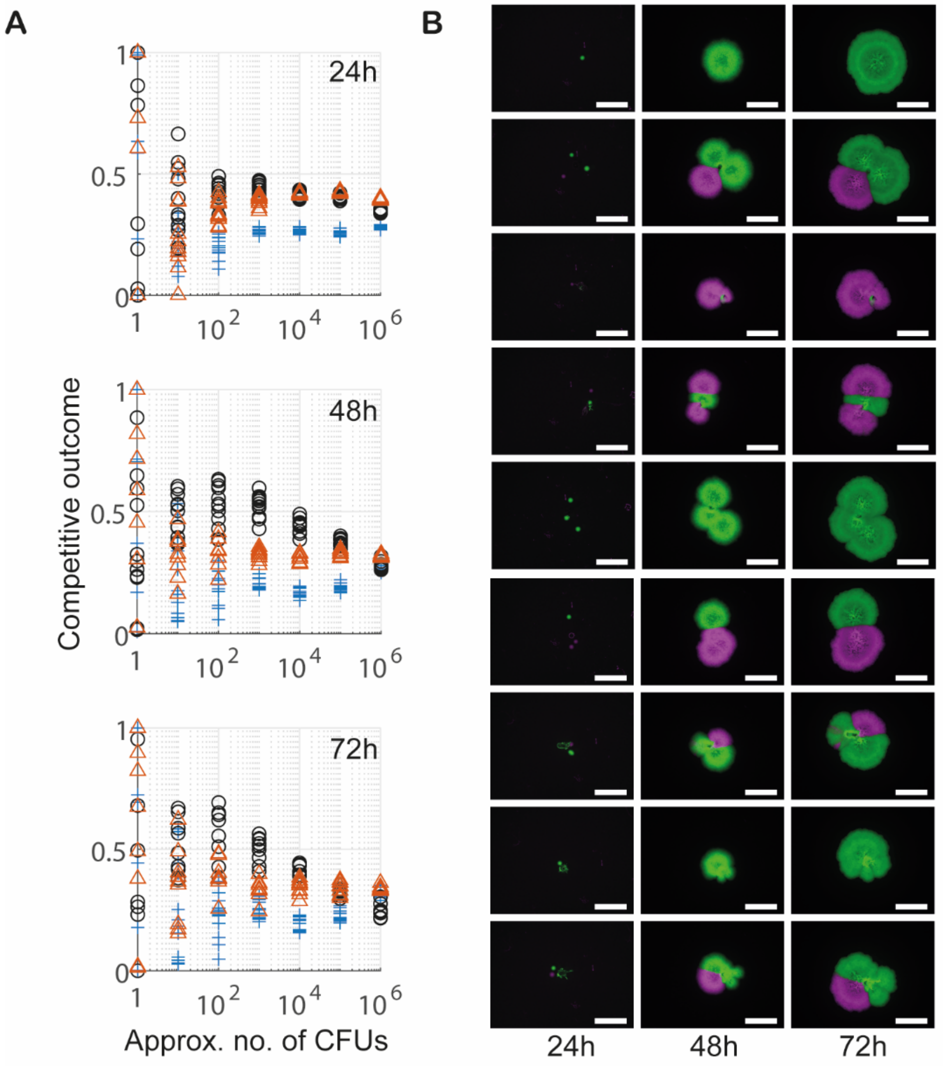

Figure S5: ***Experimental data of all three biological replicates for isogenic strain pair****.* ***A*** *Competitive outcome data for one strain only from all three biological replicates are shown for the isogenic 3610 assay at 24h, 48h and 72h after biofilm inoculation. The colour and marker shape indicate data from the same biological replicate. For any given data point, the competitive outcome of the competitor strain is the given score subtracted from unity.* ***B*** *Variability at low founder density caused by inconsistencies in initial strain ratio. Images taken after 24, 48 and 75 hours of incubation of all technical repeats within one biological repeat of our 3610 isogenic strain pair assay, inoculated with founder densities of order 1 CFU.*

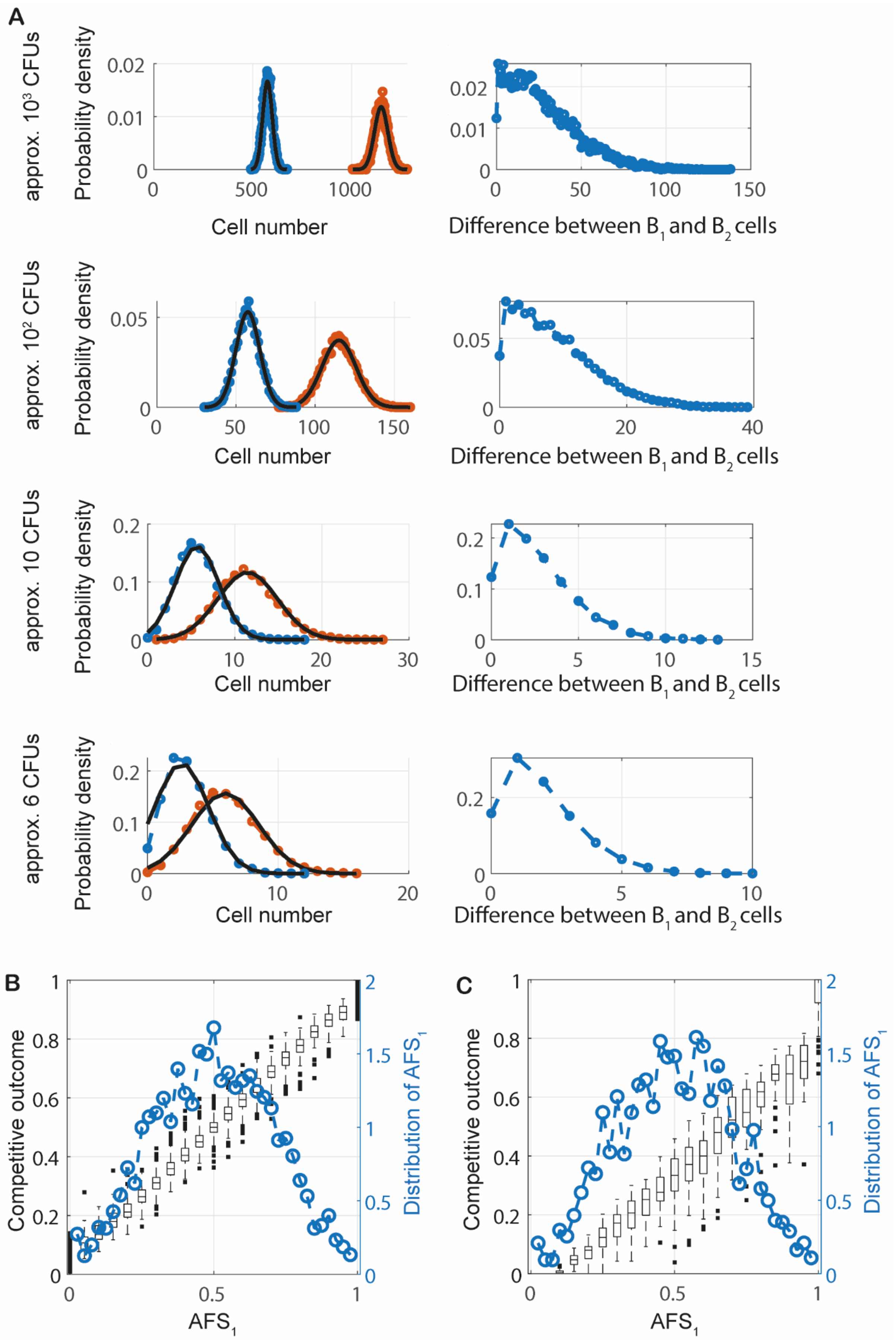

A
approx. 10^3 CFUs
approx. 10^2 CFUs
approx. 10 CFUs
approx. 6 CFUs
Probability density
Cell number
Difference between B_1 and B_2 cells
B
C
Competitive outcome
Distribution of AFS_1
AFS_1

*Figure S6: **Data from combinatorial cell picking model. A** Cell number distributions at different founder densities. Data obtained from a Monte Carlo simulation with 10000 replicates of our combinatorial cell picking model, representing the process of inoculating biofilms with 10^3, 10^2, 10 and 6 CFUs. The left column shows total cell numbers (red) and cell numbers of the strain labelled as B_1 (blue). The distribution of the second strain is the same as that for B_1. Both distributions are approximately normal, as indicated by the fitted (truncated) normal distributions (solid black curve). The right column shows the distribution of the difference between cell numbers of both strains. **B, C** Access to free space determines competitive outcome independent of initial strain ratio. The relation between the access to free space score AFS_1 and competitive outcome is shown for an isogenic strain pair (**B**) and a non-isogenic strain pair (**C**). The data are obtained from a Monte Carlo simulation (5000 realisations each), in which initial cell numbers are picked at random from the distribution calculated by our combinatorial model representing a founder density equivalent to approx. 6 CFUs to allow for comparisons with results presented in the main text. Circular makers connected by dashed lines visualise the observed distribution of AFS_1.*

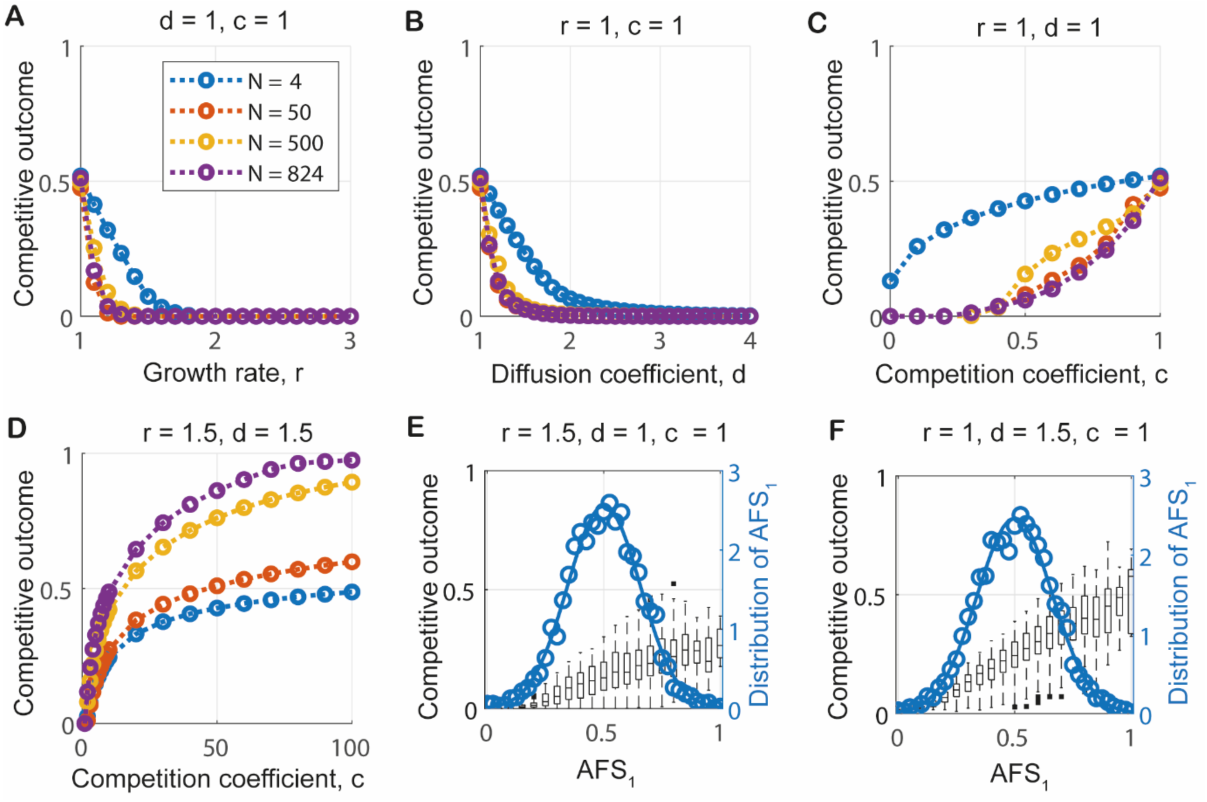

*Figure S7: **Impact of changes to model parameters on competitive outcome. A-D** The relation between competitive outcome for B_1 and the model parameters are shown. The values of the non-varying parameters are shown. For a given founder density, all simulations are performed using identical initial conditions. **E, F** The relation between access to free space and competitive outcome for B_1 is shown for two parameter sets . With the founder density fixed ($N = 6$), data are obtained from 5000 model realisations in each case. The observed probability density function for AFS is shown (circular markers) along with the density functions of fitted normal distributions ($\mu \approx 0.5, \sigma \approx 0.16$ in both) (solid lines).*

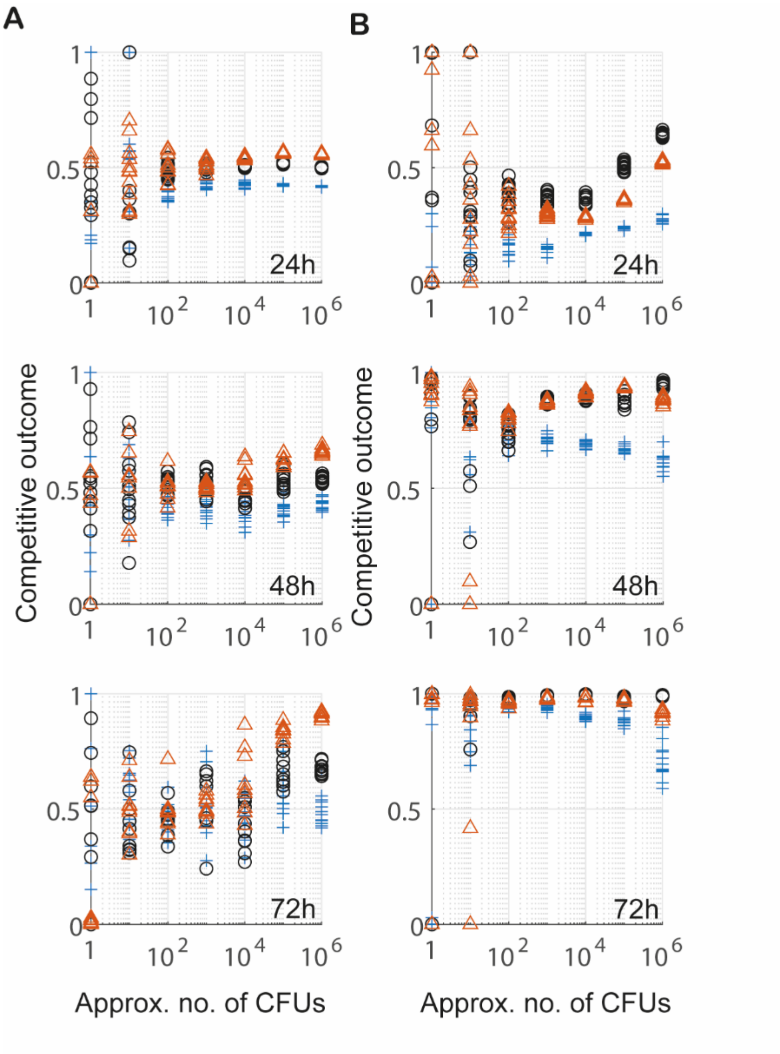

Figure S8: ***Experimental data of all three biological replicates for non-isogenic strain pair with local antagonistic interactions. A, B*** *Competitive outcome data of one strain only from all three biological replicates are shown for isogenic 6153 pair (**A**) and the competitive 3610/6153 pair (**B**) assays at 24h, 48h and 72h after biofilm inoculation. The colour and marker shape indicate data from the same biological replicate.*

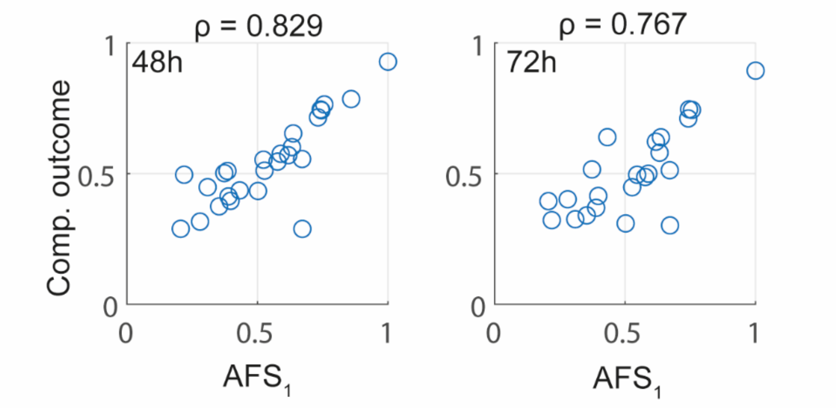

Figure S9: ***Access to free space as a predictor of competitive outcome for the isogenic 6153 pair****. The relation between access to free space, calculated based on images taken after 24h of biofilm incubation, and strain density after 48h (left,* $n = 26$*) and 72h (right,* $n = 23$*) is shown.*

S2 Supplemental movies

Movie S1: “Movie_S1_high_founder_dens_isogenic.avi”
Timelapse video of the mathematical model for isogenic strains, initialised used spatially homogeneous conditions, representing high founder density.

Movie S2: “Movie_S2_intermediate_founder_dens_isogenic.avi”
Timelapse video of one example realisation of the mathematical model for isogenic strains, initialised using $N = 824$ microcolonies, representing intermediate founder density.

Movie S3: “Movie_S3_low_founder_dens_isogenic.avi”
Timelapse video of one example realisation of the mathematical model for isogenic strains, initialised using $N = 6$ microcolonies, representing low founder density.

Movie S4: “Movie_S4_high_founder_dens_nonisogenic.avi”:
Timelapse video of the mathematical model for non-isogenic strains, initialised used spatially homogeneous conditions, representing high founder density.

Movie S5: “Movie_S5_intermediate_founder_dens_nonisogenic.avi”
Timelapse video of one example realisation of the mathematical model for non-isogenic strains, initialised using $N = 824$ microcolonies, representing intermediate founder density.

Movie S6: “Movie_S6_low_founder_dens_nonisogenic.avi”
Timelapse video of one example realisation of the mathematical model for non-isogenic strains, initialised using $N = 6$ microcolonies, representing low founder density.

S3 Models and nondimensionalisations

The mathematical model (1) in the main text is nondimensionalised. In dimensional form, it is

$$\frac{\partial \tilde{B}_1}{\partial \tilde{t}} = \nabla \cdot \left(Id \cdot \tilde{d} \left(1 - \frac{\tilde{B}_1 + \tilde{B}_2}{\tilde{k}_{cap}}\right) \nabla \tilde{B}_1 \right) + \tilde{k}_{max} \left(1 - \frac{\tilde{B}_1 + \tilde{B}_2}{\tilde{k}_{cap}}\right),$$
$$\frac{\partial \tilde{B}_2}{\partial \tilde{t}} = \nabla \cdot \left(Id \cdot \tilde{d} \left(1 - \frac{\tilde{B}_1 + \tilde{B}_2}{\tilde{k}_{cap}}\right) \nabla \tilde{B}_2 \right) + \tilde{k}_{max} \left(1 - \frac{\tilde{B}_1 + \tilde{B}_2}{\tilde{k}_{cap}}\right). \qquad (S1)$$

The variables $\tilde{B}_1(\tilde{\boldsymbol{x}}, \tilde{t})$ and $\tilde{B}_2(\tilde{\boldsymbol{x}}, \tilde{t})$ (both units: gm^{-2}) denote the densities of two bacterial strains at time $\tilde{t} > 0$ (s) and spatial position $\tilde{\boldsymbol{x}} \in \Omega$, (m^2) where $\Omega \subset \mathbb{R}^2$ is the two-dimensional spatial domain. The parameter $\tilde{k}_{max}$ (s^{-1}) is the maximum growth rate coefficient of the isogenic strains $\widetilde{B}_1$ and $\tilde{B}_2$. The carrying capacity $\tilde{k}_{cap}$ (gm^{-2}) denotes the maximum, sustainable population density. Finally, $\tilde{d}$ $(m^2 s^{-1})$ is the maximum diffusion coefficient of the strains and the indicator function $Id = 1$ if $\tilde{B}_1 + \tilde{B}_2 \leq \tilde{k}_{cap}$ and $Id = 0$ otherwise. A suitable nondimensionalisation is

$$\tilde{B}_1 = \tilde{k}_{cap} B_1, \qquad \tilde{B}_2 = \tilde{k}_{cap} B_2, \qquad \tilde{t} = \frac{1}{\tilde{k}_{max}} t, \qquad \tilde{\boldsymbol{x}} = \sqrt{\frac{\tilde{d}}{\tilde{k}_{max}}}\, \boldsymbol{x}. \qquad (S2)$$

Substitution of (S2) into (S1) yields the nondimensional model (1) in the main text.

The mathematical model (4) in the main text is nondimensionalised. In dimensional form, it is

$$\frac{\partial \tilde{B}_1}{\partial \tilde{t}} = \nabla \cdot \left(Id \cdot \tilde{d}_{\tilde{B}_1} \left(1 - \frac{\tilde{B}_1 + \tilde{B}_2}{\tilde{k}_{cap}}\right) \nabla \tilde{B}_1 \right) + \tilde{k}_{max,\tilde{B}_1} \tilde{B}_1 \left(1 - \frac{\tilde{B}_1 + \tilde{B}_2}{\tilde{k}_{cap}}\right) - \tilde{c}_{12} \tilde{B}_1 \tilde{B}_2,$$
$$\frac{\partial \tilde{B}_2}{\partial \tilde{t}} = \nabla \cdot \left(Id \cdot \tilde{d}_{\tilde{B}_2} \left(1 - \frac{\tilde{B}_1 + \tilde{B}_2}{\tilde{k}_{cap}}\right) \nabla \tilde{B}_2 \right) + \tilde{k}_{max,\tilde{B}_2} \tilde{B}_2 \left(1 - \frac{\tilde{B}_1 + \tilde{B}_2}{\tilde{k}_{cap}}\right) - \tilde{c}_{21} \tilde{B}_1 \tilde{B}_2. \qquad (S3)$$

As above, the variables $\tilde{B}_1(\tilde{\boldsymbol{x}}, \tilde{t})$ and $\tilde{B}_2(\tilde{\boldsymbol{x}}, \tilde{t})$ (both units: gm^{-2}) denote the densities of two bacterial strains at time $\tilde{t} > 0$ (s) and spatial position $\tilde{\boldsymbol{x}} \in \Omega$, (m^2) where $\Omega \subset \mathbb{R}^2$ is the two-dimensional spatial domain. The parameters $\tilde{k}_{max,\tilde{B}_1}$ and $\tilde{k}_{max,\tilde{B}_2}$ (both s^{-1}) are the maximum growth rate coefficients of B_1 and B_2, respectively. The carrying capacity $\tilde{k}_{cap}$ (gm^{-2}) denote the maximum, sustainable population density. The parameter $\tilde{c}_{12}$ $(m^2 g^{-1} s^{-1})$ denotes the amount of biomass of B_1 killed per second per unit biomass of strain B_2, and *vice versa* for $\tilde{c}_{21}$ $(m^2 g^{-1} s^{-1})$. Finally, $\tilde{d}_{\tilde{B}_1}$ and $\tilde{d}_{\tilde{B}_2}$ (both $m^2 s^{-1}$) are the maximum diffusion coefficients of $\widetilde{B_1}$ and $\widetilde{B_2}$, respectively. A suitable nondimensionalisation is

$$\tilde{B}_1 = \frac{\tilde{k}_{max,\tilde{B}_1}}{\tilde{c}_{12}} B_1, \qquad \tilde{B}_2 = \frac{\tilde{k}_{max,\tilde{B}_1}}{\tilde{c}_{12}} B_2, \qquad \tilde{t} = \frac{1}{\tilde{k}_{max,\tilde{B}_1}} t, \qquad \tilde{\boldsymbol{x}} = \sqrt{\frac{\tilde{d}_{\tilde{B}_1}}{\tilde{k}_{max,\tilde{B}_1}}}\, \boldsymbol{x},$$
$$k_{cap} = \frac{\tilde{k}_{cap} \tilde{c}_{12}}{\tilde{k}_{max,\tilde{B}_1}}, \qquad d = \frac{\tilde{d}_{\tilde{B}_2}}{\tilde{d}_{\tilde{B}_1}}, \qquad k_{max} = \frac{\tilde{k}_{max,\tilde{B}_2}}{\tilde{k}_{max,\tilde{B}_1}}, \qquad c = \frac{\tilde{c}_{21}}{\tilde{c}_{12}} \qquad (S4)$$

Substitution of (S4) into (S3) yields the nondimensional model (4) in the main text.

S4 Methods for model analysis

S4.1 Model implementation

The mathematical models were solved numerically for 25 nondimensional time units using Matlab's PDE Toolbox (Version 3.5 (Release 2020b))(1), which implements a finite element method. We chose a triangulated mesh of linear geometric order, with all mesh elements being of approximately the same size. We performed checks to ensure that the choice of mesh size did not affect results presented. The spatial domain was chosen to be large enough so that populations could not reach the boundary within the time considered in the numerical simulations. Thus, boundary conditions (the derivatives of B_1 and B_2 in the direction of the outward normal were set to zero) did not have an impact on the solution dynamics. Unless otherwise stated, parameter values were $R = 10\sqrt{50}, R_0 = 0.2R$ (both models) and $d = r = 1, c = 0.2, k = 10$ (in (4) only). The target side length of mesh elements was set to $0.125\sqrt{50}$, except in the simulations for Fig. 6D, which required a finer mesh (target side length was halved) due to the large competition coefficients.

For both PDE models, we performed model simulations for a selected set S of initial microcolony numbers, where

$$S \coloneqq \{2, 4, 6, 8, 10, 20, 30, 40, 50, 60, 70, 80, 90, 100, 120, 140, 160, 180, 200, 240, 280, 320,$$

$$360, 400, 450, 500, 600, 700, 824\}.$$

S4.2 Definition of access to free space score

The access to free space scores $AFS_i, i = 1,2$, are defined as follows. First, for a given initial condition (initial configuration), we denote by $\mathcal{B}_i = \{\boldsymbol{x} \in \Omega: B_i(\boldsymbol{x}, 0) > 0\}$ the set of loci that are assigned to microcolonies of strain B_i. To define $\mathrm{AFS_i}$, we considered a circle $\Gamma_n = \{\boldsymbol{x} \in \mathbb{R}^2: \|\boldsymbol{x} - \boldsymbol{m_c}\| = nR_0\}$, where n is a positive real number and $\boldsymbol{m_c}$ is the centre of mass of the whole initial population (Figure 3A). The number n was chosen to be sufficiently large so that Γ_n encompasses the whole of Ω_0 (i.e. $n \geq 1$). For each point $\boldsymbol{x}_c$ on the circle we (i) determined the closest microcolony, (ii) recorded which strain occupied that microcolony and (iii) assigned the point $\boldsymbol{x}_c$ to the set $\Gamma_n^{\mathcal{B}_1}$ if the closest patch belongs to $\mathcal{B}_1$ and to $\Gamma_n^{\mathcal{B}_2}$ otherwise (Fig. S3). That is

$$\Gamma_n^{\mathcal{B}_i} = \{\boldsymbol{x_c} \in \Gamma_n: \min_{y \in \mathcal{B}_i} \|\boldsymbol{x_c} - \boldsymbol{y}\| < \min_{y \in \mathcal{B}_j} \|\boldsymbol{x_c} - \boldsymbol{y}\|\}. \qquad (5)$$

The *access to free space score* of B_i, $\mathrm{AFS_i}$, was then defined to be the ratio of the total length of circle segments in $\Gamma_n^{\mathcal{B}_i}$, denoted by $\ell(\Gamma_n^{\mathcal{B}_1})$, to the circumference of the circle Γ_n i.e.

$$AFS_1 = \frac{\ell(\Gamma_n^{\mathcal{B}_1})}{2nR_0\pi}, \qquad AFS_1 = 1 - AFS_2. \qquad (6)$$

It is clear from its definition that the access to free space score depends on the choice of n, i.e. $\mathrm{AFS_i} = \mathrm{AFS_i}(n)$. It was established that even n=1 provided a reasonably accurate measure and increasing n beyond 2 did not induce a significant change in $\mathrm{AFS_i}(n)$ (Section S4.3 and Figure S8). For consistency, we used $n = 5$ in all the analysis of our modelling data, and for brevity denoted $\mathrm{AFS_i}(5)$ by $\mathrm{AFS_i}$.

We note that a Voronoi tessellation would be an alternative method of assessing a strain's potential for radial expansion (2). However, in the interest of computational efficiency, we did not consider this approach.

S4.3 Combinatorial cell picking model

To computationally model the number of cells of either strain type deposited on the agar plate during biofilm spotting, we constructed a simple combinatorial model that represents the process of extracting a small cell sample from a larger sample (e.g. extracting a 5 μl sample from 1000 μls). To mimic this process, we randomly placed points (representing bacteria) in a spherical volume of $1\ ml$ by drawing their coordinates from a uniform distribution. The number of points placed was defined by the experimentally determined number of colony forming units at each given founder density. Cells were assigned one of two types (either "magenta/B_1" or "green/B_2") at a 1:1 ratio. Then a spherical volume of $5\mu l$, representing the cell culture spotted on the growth medium, was picked at random, ensuring that it was fully contained within the original volume. The number of points of either type contained in the picked volume were recorded. This process was repeated 10000 times to obtain the distribution of founder cell ratios. While the choice of spherical volumes is not an accurate representation of the geometry of our experimental equipment, we do not expect the geometry to significantly affect the results due to the uniform distribution of points picked within the volume.

S5 Notes on variability in competitive outcome

The case of $N = 2$ which each strain only occupies one single cell patch initially is special. This initial condition induces symmetry in the system around the axis that is perpendicular to, and bisects the line connecting the initial patches. This symmetry results in identical behaviour of both strains yielding a competitive outcome of ≈ 0.5 consistently. Note the slight deviation from 0.5 which occurs due to the discretisation of the domain. It is noteworthy that this does not contradict our characterisation of competitive outcome by access to free space, discussed in the main text. If there are only two occupied cell patches initially, the system's centre of mass is the bisection point of the line connecting both patches. Hence the intersection points between the reference circle Γ_n used to define the access to free space score $\mathrm{AFS_i}$ and the system's symmetry axis are equidistant to both cell patches and split Γ_n into two segments of the same length ($\mathrm{AFS_1} = 0.5$).

The remaining small variation in the relationship between competitive outcome and AFS (Figure 3C and 3D) is generated by the necessity to bin data into finite sets and by cases for which initial founder patch configurations have identical AFS scores but generate slightly different competitive outcomes. These latter cases occur because the AFS score does not account for dynamics within the confines of the footprint of the inoculum. For example, if microcolonies of one strain are placed so they initially *encircle* those of the other, then small changes to the positions of the encircled strain's microcolonies will not change the AFS score. However, these *small* changes in initial configuration will cause *small* changes in competitive outcome. Moreover, due to the small size of the inoculum compared to the size of the mature biofilm, these small variations become negligible at larger simulation times.

S6 Model hypotheses are robust to differences in strain growth dynamics

Next, we considered the effect of allowing the growth, diffusion and competition parameters of the two strains to differ. A full sensitivity analysis that encompassed the whole parameter space for each founder density and corresponding large set of initial data was infeasible due to the high computational cost. Instead, for each founder density, we fixed an initial configuration that resulted in a typical competitive outcome (i.e. one that lied within the interquartile range given by our Monte Carlo simulations in the sense of Figure 2D). We then investigated the impact of changes to strain growth rate, competition coefficient and the diffusion coefficient.

Unsurprisingly our analysis revealed that increasing a strain's growth rate, diffusion coefficient, or competition efficiency led to an increased competitive outcome for that strain (Figure S8A,B and C). In particular, when the growth rate parameter $r \gtrsim 2$ (corresponding to a two-fold difference in strain's growth rate) or the diffusion coefficient $d \gtrsim 3$ (corresponding to a three-fold difference in strain's diffusion coefficients) coexistence was not detected for any founder density. However, the model predicted coexistence is robust over a range of differences in growth dynamics within these parameter extremes (Figure S8A and B). With strains balanced in their growth parameters, then even in the case where the weaker strain was unable to directly affect its competitor (c=0), coexistence remained possible at low founder densities (Figure S8C). For cases in which one strain was assumed to grow and diffuse 1.5 times as fast ($d = r = 1.5$) as its competitor, which in turn was assumed to be superior in its competition efficiency ($c > 1$), coexistence was generally observed (Figure S8D).

Coexistence even occurred for extreme differences in competition coefficients ($c = 100$) at lower founder densities (Figure S8D). Finally, for low founder density, model simulations confirmed that the access to free space score was robust to changes in growth and diffusion parameters and remained an accurate predictor of competitive outcome (Figure S8E and F). These analyses show that the model hypotheses are robust to differences in strain growth dynamics.

S7 Experimental methods

S7.1 Growth conditions

Bacterial strains were routinely grown on lysogeny broth (LB: 1% (w/v) Bacto-peptone, 1% (w/v) NaCl, 0.5% (w/v) yeast extract and 1.5% (w/v) agar) plates either at 37°C for 18 hours or at 22°C for 64 hours or in liquid LB broth at 37°C with agitation. For biofilm formation assays, colony forming unit counts and halo formation assays the strains were grown on MSgg agar plates (5 mM potassium phosphate (pH 7), 100 mM MOPS (pH=7), 2 mM $MgCl_2$, 700 µM $CaCl_2$, 50 µM $MnCl_2$, 50 µM $FeCl_3$, 1 µM $ZnCl_2$, 2 µM thiamine 0.5% (v/v) glycerol, 0.5% (w/v) glutamate, 1.5% (w/v) agar) (3). For growth curves, MSgg media was used in a liquid form, without the addition of agar. For transforming soil isolates, a modified version of the 10 x Modified Competency (MC) media was used (10.7 g K_2HPO_4, 5.2 g KH_2PO_4, 20 g dextrose, 0.88 g sodium citrate dehydrate, 2.2 g L-glutamic acid monopotassium salt, and 1 g tryptone per 100 ml) (4). When necessary, antibiotics were used at the following concentrations: 100 µg/ml spectinomycin, 5 µg/ml chloramphenicol and 100 µg/ml ampicillin.

S7.2 Biofilm formation assays

Strains were streaked for single colonies on LB agar plates that were incubated at 37°C overnight. A single colony was selected to inoculate 5 ml of LB broth and grown overnight at 37°C with agitation. 100 µl of the overnight culture was placed in 3 ml of LB and grown at 37°C with agitation. Once all cultures had reached or exceeded an OD_{600} of 1, incubation was stopped, and each cell culture was normalised to an OD_{600} of 1.

For determining the nature of the interaction between 3610 and 6153, three independent biological replicates were performed using strains 3610 *gfp* (NRS6942), 3610 *mTagBFP* (NRS6932), 6153 *gfp* (NRS6222) and 6153 *mTagBFP* (NRS6938). Once normalised, the cultures were mixed at a 1:1 ratio in all pairwise combinations, allowing the examination of both isogenic control pairs and non-isogenic biofilm outcomes. Three technical replicated were performed for each combination and each biological repeat.

For examining the impact of different founder densities on competitive outcomes, three independent biological repeats were performed, all including isogenic mixed biofilms of both backgrounds (3610 *mTagBFP* mixed with 3610 *gfp* and 6153 *mTagBFP* mixed with 6153 *gfp*). For the non-isogenic mixes, two rounds were performed in which 3610 *gfp* was mixed with 6153 *mTagBFP* and one round was performed with the fluorescent markers swapped around (3610 *mTagBFP* mixed with 6153 *gfp*). The mixed cultures were 10-fold serial diluted and twelve technical replicates were performed for each founder density. To establish colony biofilms, 5 µl of cell culture was placed onto MSgg agar plates. Once dried, the plates were incubated at 30°C and imaged using a Leica MZ16 FA stereoscope and LAS version 2.7.1 and/ or fixed for flow cytometry after 24, 48, or 72 hours of incubation (see below). Imaging data was imported to OMERO for archiving and figure construction (5).

S7.3 Determining cell counts

To calculate the number of colony forming units in the culture at an OD_{600} of 1, strains were grown and the OD_{600} normalised as described above. The prepared cultures were serially diluted, 100 µl of the 10^{-5} and 10^{-6} dilutions spread onto MSgg agar plates and incubated at 30°C for 48 hours. Samples containing between 30 and 300 colonies were used for determining the number of colony forming units per ml of culture.

S7.4 Single cell analysis by flow cytometry

Biofilms grown as described above were removed from the agar plates using sterile loops, placed into 500 µl of GTA buffer (10 mM EDTA (pH=8), 20mM Tris-HCl (pH=8), 50mM glucose) and disrupted by passing through a 23-gauge needle to disrupt the biofilms before centrifuging at 17,000 x *g* for 5 minutes. The supernatant was removed, the pelleted cells were suspended in 500 µl of 4% (w/v) formaldehyde and incubated at room temperature for 7 minutes. The tubes were centrifuged at 17,000 x *g* for 5 minutes, the formaldehyde was discarded, and the samples washed by suspending the pellet in 500 µl of GTA buffer, centrifuging for 5 minutes and removing the supernatant. The pellets were resuspended in 500 µl of GTA buffer and the samples stored at 4°C. Prior to flow cytometry, the samples were mildly sonicated and 0.1 – 1 µl of the cell sample added to 1 ml of PBS buffer supplemented with 0.5% (w/v) BSA. Flow cytometry was performed using a LSRFortessa™ (BD biosciences) instrument. The results were analysed using Flowjo (version 10.7.1) to extract the % of cells expressing GFP and mTagBFP in each sample. Figures were constructed using GraphPad prism 7.

S7.5 Antibiosis halo assays

Strains were grown on LB agar plates. Single colonies were used to establish cultures in 5 ml of LB that were grown at 37°C with agitation overnight. 100 µl of the overnight cultures was placed in 3 ml of LB and grown at 37°C with agitation for 3 hours. At this point the cultures were normalised to a standard OD_{600} of 0.5. For the lawn (target), 1 µl of each normalised culture was mixed with 100 µl of LB that spread across the surface of an MSgg plate. For the spot (attacker), 6 µl of the normalised cultures was placed in the centre of the agar where the target cells had been spread. The plates were left at room temperature for an hour to dry and incubated at 30°C. The cells were imaged after 24 and 48 hours using a using a Leica MZ16 FA stereoscope and LAS version 2.7.1. The imaging data was uploaded to OMERO (5) for figure construction.

S7.6 Growth curves

NCIB 3610 and NRS6153 were grown on LB agar plates at 37°C overnight to obtained single colonies. The following day, 5 ml cultures of LB broth were inoculated with single colonies and incubated at 37°C overnight with gentle agitation. 100 µl of the overnight cultures was added to 3 ml of LB broth and grown at 37 °C with agitation for 4 hours. The bacteria were harvested by centrifugation and suspended in 1 ml of MSgg media. The OD_{600} was measured and normalising to an OD_{600} of 1. The cell suspension was used to inoculate the growth curve cultures with a starting OD_{600} of 0.01 in 25 ml of MSgg. These cultures were growth for 29 hours in a 30°C water bath and OD_{600} measurements were taken every 2 hours over that period. The generation times were calculated by plotting the exponential phase of each growth curve and fitting an exponential trend-line which was used to resolve the generation time. 3 biological repeats were conducted and the average generation time for each strain was calculated. Growth curve data was plotted in GraphPad Prism 7.

S8 *Bacillus subtilis* strains

S8.1 Strain construction

All *B. subtilis* strains used in this study are listed in Table S1. Plasmid pNW2303 is derived from pUC57 and carries the *mTagBFP* coding sequence (Table S2). The *mTagBFP* sequence was introduced by restriction enzyme digest to the pDR111 vector generating plasmid pNW2304 and allowing integration into the *amyE* locus. pDR111 carries the P*hyperspank* (P_{IPTG}) promoter and removal of the *lacI* gene by insertion of the *mTagBFP* sequence using HindIII and BamHI allows for constitutive expression. *E. coli* strain MC1061 was used for cloning purposes (*F'lacIQ lacZM15 Tn10 (tet)*).

For construction of NCIB 3610 derivatives expressing *gfpmut2* and *mTagBFP* respectively, plasmids pBL165 (containing the *gfpmut2* sequence flanked by the *amyE* locus, linked to a chloramphenicol resistance cassette) (6) and pNW2304 were integrated into the *B. subtilis* 168 genome (7). Homologous recombination at the *amyE* locus was confirmed using a potato starch assay (8). Each allele was introduced into the NCIB 3610 genome using SPP1 phage transduction (9).

For transformation of the soil isolate NRS6153 natural genetic competency was induced as described previously (4). Briefly, a day culture of each isolate was established in 2 ml of 1x MC media supplemented with 3 mM $MgSO_4$ and 875 µM $FeCl_3$. The cultures were grown for 4.5 hours at 37°C with gentle agitation. Approximately 25 µg of pBL165 or pNW2304 was added to 400 µl of cells and the reaction was incubated at 37°C with agitation for a further 90 minutes. The cells were plated onto LB agar containing the appropriate antibiotics for selection (chloramphenicol for pBL165 or spectinomycin for pNW2304). The resulting colonies were screened as described above to ensure integration at the *amyE* locus.

S8.2 Strain table

**Strain**	**Relevant genotype** [a]	**Source/ construction** [b,c]
NCIB 3610	Wild type, prototroph	BGSC
168	*trpC2*	BGSC
NRS6153	Wild type, prototroph	(10)
NRS6222	NRS6153 *amyE::*P*hyperspank- gfpmut2 (cml)*	pBL165 → NRS6153
NRS6900	168 *trpC2 amyE::*P*hyperspank- gfpmut2 (cml)*	pBL165 → 168
NRS6931	168 *trpC2 amyE::*P*hyperspank- mTagBFP (spec)*	pNW2304 → 168
NRS6932	NCIB 3610 *amyE::*P*hyperspank- mTagBFP (spec)*	SPP1 NRS6931 → NCIB3610
NRS6938	NRS6153 *amyE::*P*hyperspank- mTagBFP (spec)*	pNW2304 → NRS6153
NRS6942	NCIB 3610 *amyE::*P*hyperspank- gfpmut2 (cml)*	SPP1 NRS6900 → NCIB 3610

Table S1: List of *B. subtilis* strains used in this study

[a.] Antibiotic resistance cassettes are as follows; "spec" indicates a spectinomycin resistance cassette and "cml" indicates a chloramphenicol resistance cassette

[b.] BSGC is the *Bacillus* genetic stock centre

[c.] The arrows show the direction of the strain construction where SPP1 phage or plasmid DNA (left of the arrow) are inserted into a recipient strain (right of the arrow)

S9 Sequence of *mTagBFP* coding region

***mTagBFP* construct sequence (5’-3’)** [a]
**AAGCTT**AAGGAGGTGATCATTAAAA*ATGAGCGAACTGATCAAAGAAAACATGCATATGAAACTGTACATG*<br>*GAAGGCACAGTCGATAACCATCACTTTAAATGCACATCAGAAGGCGAAGGCAAACCGTATGAAGGCACAC*<br>*AAACAATGAGAATCAAAGTTGTTGAAGGCGGACCGCTGCCGTTTGCATTTGATATTCTGGCAACATCATTTC*<br>*TGTATGGCAGCAAAACGTTTATCAATCATACACAAGGCATCCCGGATTTTTTTAAACAATCATTTCCGGAAG*<br>*GCTTTACATGGGAACGCGTTACAACATATGAAGATGGCGGAGTTCTGACAGCAACACAAGATACATCATTG*<br>*CAAGATGGCTGCCTGATCTATAATGTCAAAATTAGAGGCGTCAACTTTACAAGCAATGGCCCTGTTATGCAG*<br>*AAAAAAACACTGGGCTGGGAAGCATTTACAGAAACACTGTATCCGGCTGATGGCGGACTGGAAGGCAGAA*<br>*ACGATATGGCACTGAAACTGGTTGGCGGATCACATCTGATTGCAAACATCAAAACAACGTACCGCTCAAAA*<br>*AAACCGGCAAAAAATCTGAAAATGCCTGGCGTCTATTATGTCGATTATAGACTGGAACGCATCAAAGAAGC*<br>*GAACAACGAAACATATGTCGAACAACATGAAGTTGCAGTTGCGAGATATTGCGATCTGCCGTCAAAACTGG*<br>*GCCATAAACTGAATTA***GGATCC**

Table S2: Sequence of *mTagBFP* coding region

[a] The restriction sites are shown in bold, the ribosome binding site and linker sequence are underlined, and the mTagBFP coding region is in italics.

S10 Image analysis

We determine relative strain densities in competitive biofilm assays using data obtained through image analysis on the total intensity of a fluorescent signal in pixels where the signal is above the background threshold. To achieve this a Fiji/ImageJ (11, 12) macro was written (13). Since images were saved as multi-series Leica LIF files this macro relies on Bio-Formats Macro Extensions (14) to import the data. The macro can perform batch analysis of all images in a file, writing a summary table of results in CSV format as well as snapshot TIFF images showing detected biofilm regions as overlay outlines. The macro uses built-in functionality of ImageJ to detect biofilm regions, specifically: auto-thresholding using the "Triangle" method after optional background subtraction using the rolling ball / sliding paraboloid algorithm. A single large colony biofilm in the centre of the image or several smaller "sub-colonies" can optionally be detected (the former using the "Wand" tool, the latter using "Analyze Particles" with a specified size range). For the biofilm region in each image the following measurements are made for 2 channels: area, basic intensity statistics (mean, maximum, standard deviation, total intensity) and some colocalization statistics (Pearsons Correlation Coefficient, thresholded "Object Pearsons" (15) and optionally Manders M1 and M2 coefficients (16). Finally, percentage area within the biofilm region that is above background for each channel is reported, as well as total "foreground" signal (i.e. total signal in pixels that have above-background intensity values). These final two measurements rely on a background intensity parameter for each channel that distinguishes positive expression of the label from background.

For each image, the relative strain density was calculated by dividing the strain's foreground signal by the total foreground signal in the image.

S10.1 Access to free space

To calculate the access to free space score from experimental data, we used the same method as in our classification of the model initial conditions. We chose the reference circle to be of radius $R \approx 12\ mm$. The radius is an approximation as we used the number of pixels as a proxy unit for length and pixel sizes slightly varied between images. The access to free space score in our experimental assays was calculated based on images taken after 24h of incubation. Only cultures in which microcolonies did not overlap at this time point are analysed. For each image, two subimages showing the signal of one fluorescent signal only were converted into binary images using colour segmentation based on Delta E colour difference. For each pixel of the image, these binary images provided information on which strain (if any) occupies the space shown in the pixel. This analysis determines the locations of the microcolonies and the access to free space score was calculated by the same means as for the mathematical model ((17), see also Section S4.2).

S11 References

1. The MathWorks Inc. Partial Differential Equation Toolbox™ User's Guide. 2020.
2. Lloyd DP, Allen RJ. Competition for space during bacterial colonization of a surface. J R Soc Interface. 2015;12(110):0608.
3. Branda SS, Gonzalez-Pastor JE, Ben-Yehuda S, Losick R, Kolter R. Fruiting body formation by *Bacillus subtilis*. Proc Natl Acad Sci U S A. 2001;98(20):11621-6.
4. Konkol MA, Blair KM, Kearns DB. Plasmid-encoded ComI inhibits competence in the ancestral strain of Bacillus subtilis. Journal of Bacteriology. 2013.
5. Allan C, Burel JM, Moore J, Blackburn C, Linkert M, Loynton S, et al. OMERO: flexible, model-driven data management for experimental biology. Nat Methods. 2012;9(3):245-53.
6. Stanley NR, Britton RA, Grossman AD, Lazazzera BA. Identification of catabolite repression as a physiological regulator of biofilm formation by *Bacillus subtilis* by use of DNA microarrays. J Bacteriol. 2003;185(6):1951-7.
7. Harwood CR, Cutting SM. Molecular biological methods for *Bacillus*. John Wiley &Sons Ltd. Chichester, England. 1990.
8. Gillespie RM, Stanley-Wall NR. Enzymes in action: an interactive activity designed to highlight positive attributes of extracellular enzymes synthesized by microbes. Journal of microbiology & biology education. 2014;15(2):310-2.
9. Verhamme DT, Kiley TB, Stanley-Wall NR. DegU co-ordinates multicellular behaviour exhibited by Bacillus subtilis. Mol Microbiol. 2007;65(2):554-68.
10. Kalamara M, Abbott JC, MacPhee CE, Stanley-Wall NR. Biofilm hydrophobicity in environmental isolates of *Bacillus subtilis*. Microbiology (Reading). 2021;167(9).
11. Schneider CA, Rasband WS, Eliceiri KW. NIH Image to ImageJ: 25 years of image analysis. Nat Methods. 2012;9(7):671-5.
12. Schindelin J, Arganda-Carreras I, Frise E, Kaynig V, Longair M, Pietzsch T, et al. Fiji: an open-source platform for biological-image analysis. Nat Methods. 2012;9(7):676-82.
13. Eigentler L, Ball G. Code for Eigentler et al. 2021 Founder cell configuration drives competitive outcome within colony biofilms Zenodo 2021; DOI: 10.5281/zenodo.5041981
14. Linkert M, Rueden CT, Allan C, Burel JM, Moore W, Patterson A, et al. Metadata matters: access to image data in the real world. J Cell Biol. 2010;189(5):777-82.
15. Barlow AL, Macleod A, Noppen S, Sanderson J, Guerin CJ. Colocalization analysis in fluorescence micrographs: verification of a more accurate calculation of pearson's correlation coefficient. Microsc Microanal. 2010;16(6):710-24.
16. Manders EM, Stap J, Brakenhoff GJ, van Driel R, Aten JA. Dynamics of three-dimensional replication patterns during the S-phase, analysed by double labelling of DNA and confocal microscopy. Journal of Cell Science. 1992;103(3):857-62.
17. Founder cell configuration drives competitive outcome within colony biofilms. BioStudies. 2021. Available from: https://www.ebi.ac.uk/biostudies/studies/S-BSST676.
